# FOXO3-mediated chemo-protection in high-stage neuroblastoma depends on wild-type TP53 and SESN3

**DOI:** 10.1038/onc.2017.288

**Published:** 2017-09-04

**Authors:** M Rupp, J Hagenbuchner, B Rass, H Fiegl, U Kiechl-Kohlendorfer, P Obexer, M J Ausserlechner

**Affiliations:** 1 Department of Pediatrics II, Medical University Innsbruck, Innsbruck, Austria; 2 Department of Pediatrics I, Medical University Innsbruck, Innsbruck, Austria; 3 Tyrolean Cancer Research Institute, Innsbruck, Austria; 4 Department of Obstetrics and Gynecology, Medical University Innsbruck, Innsbruck, Austria

## Abstract

Forkhead box O class transcription factors are homeostasis regulators that control cell death, longevity and therapy-resistance. In neuroblastoma (NB), nuclear FOXO3 correlates with stage M disease and poor prognosis. To analyze whether FOXO3 contributes to drug-resistance in this childhood cancer, we investigated how different high-stage-derived NB cells respond to the activation of an ectopic FOXO3 allele. We found endogenous FOXO3 mostly localized to the nucleus—upon activation of an ectopic, 4OHT-activated FOXO3(A3)ER fusion protein two of the cell lines underwent apoptosis, whereas in the others FOXO3-activation even increased survival during drug-treatment. In the latter cell type, FOXO3 did not induce the BH3-only protein BCL2L11/BIM due to impaired binding of FOXO3 to the *BIM*-promoter, but still activated other FOXO3 targets. It was shown before that FOXO3 and TP53 physically interact with each other at two different regions—the TP53-N-terminus binds to the FOXO3-DNA binding domain (DBD) and the FOXO3-C-terminus interacts with the TP53-DBD. Interestingly, cell lines that undergo FOXO3-induced cell death carry homozygous point mutations in the TP53-DBD near the structural hotspot-mutation-site R175H, which abrogated FOXO3–TP53 interaction. In contrast, in FOXO3-death-resistant cells no point mutations in the TP53-DBD were found—in these cells FOXO3–TP53 complexes are formed and FOXO3-binding to the *BIM*-promoter, but not the induction of the detoxifying protein SESN3, were prevented, which in turn increased chemo-protection in this type of high-stage-derived NB cells. Our combined data suggest that FOXO3 steps in as a death inducer in case of TP53-mutation, whereas functional TP53 alters FOXO3-target-promoter-recognition, which prevents death induction by FOXO3 and instead increases chemo-protection and survival of NB cells. This novel mechanism may explain the low incidence of TP53 mutation in high-stage NB at diagnosis and suggests FOXO3 as a therapeutic target for this childhood malignancy.

## Introduction

The mammalian forkhead box O (FOXO) transcription factor family consists of four members, FOXO1/FKHR, FOXO3/FKHRL1, FOXO4/AFX and FOXO6, which are involved in multiple cellular processes ranging from apoptosis induction to longevity.^1^ FOXO3-mediated cell death in neuroblastoma (NB), an aggressive childhood tumor that develops form precursor cells of the neural crest during embryogenesis,^2^ has been associated with induction of the pro-apoptotic BH3-only proteins B-cell-lymphoma-gene-2-like-11 (BCL2L11/BIM), phorbol-12-myristate-13-acetate-induced-protein-1 (PMAIP1/NOXA) and repression of pro-survival BCL2-like-1 (BCL2L1/BCLXL) and the baculoviral-inhibitor-of-apoptosis-repeat-containing-5 (BIRC5/survivin) and is modulated by the ROS-regulating chromosome-10-open-reading-frame-10-protein (C10ORF10/DEPP).^3, 4, 5, 6, 7^ FOXO3-activity is mainly regulated by post-translational modifications (reviewed in Ausserlechner *et al.*^8^ and Hagenbuchner and Ausserlechner^9^), resulting in changes of its cellular localization or affecting its binding to DNA or proteins. Protein-kinase-B (PKB) which is frequently hyper-activated in high-stage NB due to aberrant expression of the brain-derived-neurotrophic-factor and its cognate receptor NTRK2/TRKB^10^ directly phosphorylates FOXO3 causing association with 14-3-3 proteins, nuclear exclusion and transcriptional inactivation of FOXO3 in malignant NB cells.^5, 11, 12, 13^ We previously demonstrated that in patient biopsies FOXO3 localizes to the nucleus even when phosphorylated at the PKB-site threonine-32.^14^ Stress conditions, such as chemotherapy, override growth-factor-mediated phosphorylation/inactivation of FOXO3 by PKB, which results in the relocation of FOXO3 to the nucleus.^3, 9^

FOXO3 was initially considered as a tumor suppressor by inducing apoptosis or cell-cycle-arrest.^15^ However, the opposed function of FOXO3 in cellular detoxification,^6, 16^ the development of drug-resistance^17, 18^ and the feedback-regulation on PKB-activity^19^ uncovered an additional, tumor-promoting role of FOXO3.

Beside post-translational modifications FOXO3’s activity can also be influenced by co-factor binding. One binding partner of FOXO3 is the tumor protein TP53/p53 which is mutated in over 50% of cancer types.^20^ In NB, however, mutations of TP53 are rare at diagnosis (less than 2% of patients), but frequent loss of function is observed in relapsed tumors.^21^ FOXO3 and TP53 share numerous target genes such as CDKN1/P21CIP1, GADD45A or BBC3/PUMA; hence, they are involved in the same cellular processes like cell-cycle-arrest, DNA-damage-repair and apoptosis.^22, 23, 24, 25^

In this study, we discovered that in patient-derived, high-stage NB cells that express wild-type TP53, FOXO3 interacts with TP53, which prevents the binding of FOXO3 to the *BIM*-promoter and FOXO3-induced cell death. In these cells, FOXO3-activation even enhances the resistance to chemotherapy via increased expression of SESN3.

## Results

### High-stage NB cell lines differentially respond to active FOXO3

**Figure 1:**
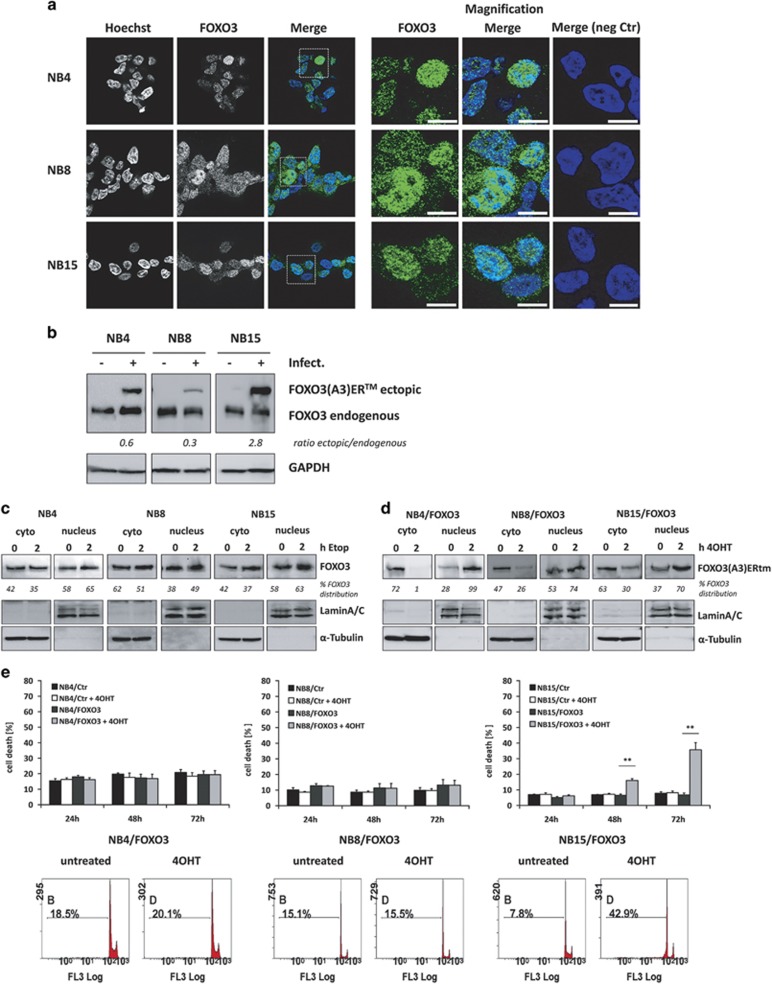
FOXO3 localizes to the nucleus of high-stage NB cell lines. (**a**) NB4, NB8 and NB15 cells were fixed with 4% ROTI-Histofix and stained with an antibody against FOXO3. Nuclei were visualized with Hoechst33342 dye. Images were acquired by an Axiovert200M microscope equipped with an ApoTome.2. Bar is 10 μm. (**b**) Ectopic FOXO3 expression in cells infected with the pLIB-FOXO3(A3)ERtm-Neo vector. (**c**) NB4, NB8 and NB15 or (**d**) NB4/FOXO3, NB8/FOXO3 and NB15/FOXO3 cells were treated with etoposide (10 μg/ml) or 100 nM 4OHT, respectively, for 2 h. Cytoplasmatic and nuclear fractions were separated by immunoblot and incubated with an antibody against FOXO3. LaminA/C (nuclear) and α-Tubulin (cytoplasmic) served as loading controls. (**e**) NB4/Ctr, NB4/FOXO3, NB8/Ctr, NB8/FOXO3, NB15/Ctr and NB15/FOXO3 cells were treated with 50 nM 4OHT for 24, 48 and 72 h and subjected to cell death analyses by PI-FACS analyses. Shown are representative images and means ±s.e.m. of three independent experiments. **P<0.01.

FOXO transcription factors are substrates of the phosphatidylinositol3-kinase–PKB-signaling-pathway and phosphorylation by PKB triggers nuclear export and functional inactivation of these transcription factors (reviewed in Arden and Biggs^26^). We demonstrated before that in NB cells FOXO3 is frequently PKB-phosphorylated and that especially in high-stage-derived NB cells FOXO3 partially localizes to the nucleus despite highly active PKB (Figures 1a and c, Supplementary Figures S1a and c).^14, 27^ In these cells, oxidative stress and DNA-damage further increase the amount of nuclear FOXO3, suggesting that the cell-stress-dependent regulation of FOXO3 is still functional (Figure 1c). To further analyze FOXO3-function in high-stage NB cells, we used an ectopically expressed 4OHT-inducible, PKB-phosphorylation-independent FOXO3(A3)ER fusion-protein and infected high-stage NB1, NB3, NB4, NB8 and NB15 cells.^14, 28^ The expression and translocation of ectopic FOXO3 after 4OHT-treatment was verified by immunoblot (Figures 1b and d and Supplementary Figure S1b). Also, this protein partially localizes to the nucleus in absence of 4OHT, indicating PKB-independent FOXO3-regulation in such high-stage-derived NB cells. Activation of the ectopic FOXO3 allele by 4OHT induces cell death in NB15/FOXO3 and NB3/FOXO3 cells, but not in NB1/FOXO3, NB4/FOXO3 or NB8/FOXO3 cells (Figure 1e and Supplementary Figure S2). In NB15/FOXO3 cells, treatment with 4OHT for 72 h increased apoptosis up to 40% compared to control cells (NB15/Ctr, Figure 1e). Within 24 h of 4OHT-treatment, NB15/FOXO3 cells arrest in G1-phase of the cell-cycle prior to cell death-induction, whereas cell-cycle progression was neither affected in NB4/FOXO3 nor in NB8/FOXO3 cells upon 4OHT-treatment (Supplementary Figure S2b). These findings demonstrate that high-stage NB cell lines vary in their physiological response to FOXO3-activation.

### BIM-regulation is impaired in FOXO3-resistant high-stage NB cell lines

**Figure 2:**
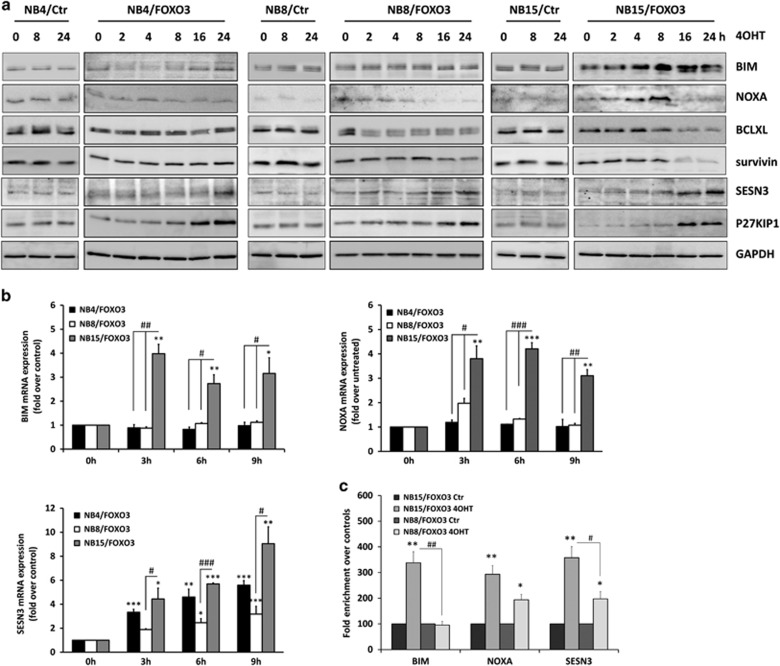
FOXO3 response correlates with differential regulation of FOXO3 target genes. (**a**) Lysates of NB4/FOXO3, NB8/FOXO3, NB15/FOXO3, and their corresponding control cells treated with 50 nM 4OHT for 0, 2, 4, 8, 16 and 24 h were subjected to immunoblot analyses using antibodies specific for BIM, NOXA, BCLXL, survivin, SESN3 and P27KIP1. GAPDH was used as loading control. (**b**) BIM, NOXA and SESN3 mRNA levels were measured by quantitative RT–PCR in NB4/FOXO3, NB8/FOXO3 and NB15/FOXO3 cells after treatment with 100 nM 4OHT for 0, 3, 6 and 9 h. Bars represent±s.e.m. of three independent experiments, each performed in triplicates. Significantly different to untreated cells:***P<0.001, **P<0.01, *P<0.05; significant differences between 4OHT-treated cells: ^###^*P*<0.001, ^##^*P*<0.01, ^#^*P*<0.05. (**c**) ChIP analyses were performed with NB8/FOXO3 and NB15/FOXO3 cells treated with 100 nM 4OHT for 6 h. Binding of FOXO3 to the promoter regions of *BIM, NOXA* and *SESN3* was quantified by quantitative PCR. Shown is the mean value±s.e.m. of three independent experiments, each performed in duplicates. Significantly different to untreated cells: **P<0.01, *P<0.05; significant differences between 4OHT-treated cells: ^##^*P*<0.01, ^#^*P*<0.05. ChIP, chromatin-immunoprecipitation

In NB, FOXO3-induced cell death depends on the induction of the pro-apoptotic BH3-only proteins BIM and NOXA, the repression of the pro-survival BCL2-protein BCLXL and the inhibitor-of-apoptosis-protein survivin.^3, 4, 5^ Expression analysis of these proteins in FOXO3-resistant NB4/FOXO3 and NB8/FOXO3 and FOXO3-sensitive NB15/FOXO3 cells as well as in their corresponding controls revealed that in NB15/FOXO3 cells BIM and NOXA strongly increased^5^ on protein- and mRNA-level, and BCLXL and survivin were repressed (Figures 2a and b and Supplementary Figure S3). In resistant NB4/FOXO3 or NB8/FOXO3 cells, however, BIM was not regulated at all. A slight repression of BCLXL and survivin was observed in NB8/FOXO3 cells on protein-, and in NB4/FOXO3 on mRNA-level (Figure 2a and Supplementary Figure S3), suggesting that induction of pro-apoptotic BIM might induce a feed-forward-regulation, for example via accumulation of ROS that further increases target-gene-regulation and drives cells into death. The ROS-detoxifying enzyme SESN3 and the cell-cycle-regulator CDKN1B/P27KIP1, both *bona-fide* targets of FOXO3,^29, 30^ were induced in all three cell lines, which is also a proof that the ectopically expressed FOXO3 allele is active and functional (Figures 2a and b).

To analyze whether the lack of transcriptional regulation of apoptosis-regulatory targets might be related to impaired promoter-binding, we performed chromatin-immunoprecipitation experiments and investigated whether FOXO3 differentially binds to the *BIM-, NOXA*- and *SESN3*-promoter in NB15/FOXO3 and NB8/FOXO3 cells. Activation of ectopic FOXO3 increased the amount of FOXO3 at the *BIM*-promoter more than threefold in NB15/FOXO3 cells, whereas no changes in FOXO3-binding were detected in NB8/FOXO3 cells (Figure 2c). In contrast, in both cell lines significantly increased FOXO3-binding to the *SESN3*-promoter was detectable (Figure 2c), correlating with mRNA and protein levels (Figures 2a and b) and also FOXO3-binding to the *NOXA-*promoter was increased in both cell lines (Figure 2c). This suggests that in resistant NB8/FOXO3 cells FOXO3 does not bind to the *BIM*-promoter upon activation, but still activates other transcriptional targets.

**Figure 3:**
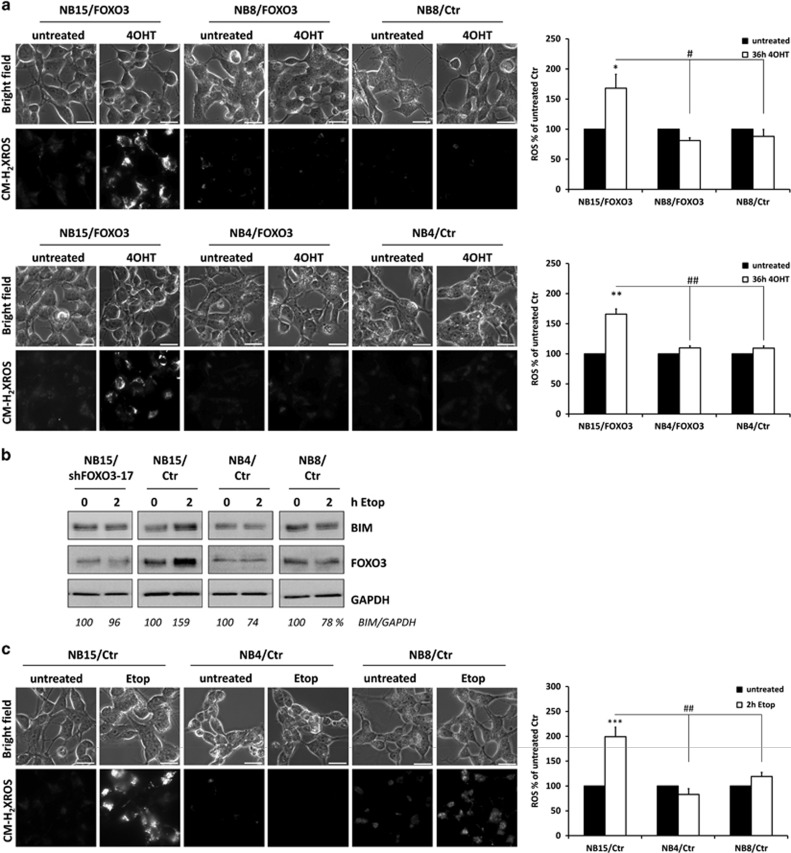
Induction of ROS accumulation by FOXO3 or etoposide correlates with death sensitivity. (**a**) NB15/FOXO3, NB8/FOXO3 and NB4/FOXO3 cells were treated with 50 nM 4OHT for 36 h. ROS accumulation was analyzed using CM-H_2_XROS. Images were acquired by live-cell imaging using an Axiovert200M microscope, equipped with a × 63 oil objective, bar size is 20 μm. Densitometry was performed using AxioVision software version 4.8; significantly different to untreated cells: **P<0.01, *P<0.05; significantly different between 4OHT-treated cells: ^##^*P*<0.01, ^#^*P*<0.05. (**b**) NB15/shFOXO3-17, NB15/Ctr, NB4/Ctr and NB8/Ctr cells were treated with 10 μg/ml etoposide for 0 and 2 h and cell lysates were subjected to immunoblot analyses using antibodies specific for BIM and FOXO3. GAPDH was used as loading control. Densitometry analyses were performed using Labworks software version 4.5 (UVP, UK). (**c**) NB15/Ctr, NB4/Ctr and NB8/Ctr cells were treated for 2 h with 10 μg/ml etoposide. ROS accumulation was analyzed using CM-H_2_XROS. Bar size is 20 μm. Densitometric analyses were performed using AxioVision software version 4.8; significantly different to untreated ***P<0.001, significantly different between 4OHT treated ^##^*P*<0.01.

We demonstrated previously that BIM-induction by FOXO3 triggers a biphasic ROS-accumulation, which critically regulates cell death-induction by FOXO3-activation and/or by addition of DNA-damaging agents in NB.^3, 31^ The primary ROS-wave is detected after 12 h *post* FOXO3-activation, the second, much more pronounced ROS-wave reaches a climax between 36 and 48 h after FOXO3-activation in NB15/FOXO3 cells.^3^ We therefore investigated, whether FOXO3-resistant NB4/FOXO3 and NB8/FOXO3 cells show comparable ROS-accumulation or whether this ROS-burst is absent in the resistant cell lines. As shown in Figure 3a, neither in NB4/FOXO3 nor in NB8/FOXO3 cells an induction of ROS was detected after 36 h, which correlated with the lack of BIM-induction (Figures 2a and b) in response to FOXO3-activation. We demonstrated before that DNA-damaging agents, at least in part trigger apoptotic cell death via a FOXO3-BIM-ROS pathway in NB cells. To analyze whether DNA-damage causes the primary ROS-wave also in resistant NB cells these cells were treated with etoposide and BIM steady-state expression as well as ROS-levels were analyzed (Figures 3b and c). Consistent with lack of BIM-induction by direct activation of FOXO3 in resistant cells (Figure 2a), etoposide-treatment induced BIM only in NB15 cells, but not in NB4 or NB8 cells (Figure 3b). As a control for the relevance of FOXO3 in this process, we included NB15/shFOXO3-17 cells with constitutive knockdown of FOXO3 by shRNA-expression. In these cells, induction of BIM by etoposide (Figure 3b) and ROS accumulation^3^ is completely prevented, proving that etoposide leads to induction of BIM and further ROS via FOXO3. ROS-levels, as measured by MitoTrackerRed (CM-H_2_XROS) staining, were markedly induced in NB15 cells, completely absent in NB4 cells and only a faint, statistically not significant increase was observed in NB8 cells upon etoposide treatment, correlating with the lack of BIM regulation in the resistant cells. Taken together our results suggest that resistance to FOXO3-induced apoptosis in high-stage NB cells correlates with the absence of BIM-induction.

Promoter-binding of transcription factors can be influenced by promoter-methylation, post-translational modifications or interaction with distinct co-factors. To analyze whether one of these conditions affects FOXO3-binding to the *BIM*-promoter, we performed MethyLight PCR in cells treated with 4OHT and 5-Aza-2′-deoxycytidine (5-azadC, positive control). However, we did not detect any DNA-methylation of the classical *BIM-*promoter region in NB4/FOXO3, NB8/FOXO3 and NB15/FOXO3 cell lines compared to a methylated control gene (data not shown) and an alternative *BIM*-promoter (BIM_02; described in Gaviraghi *et al.*^32^) was only methylated in NB8/FOXO3 and NB15/FOXO3 cells, but not in NB4/FOXO3 cells (Supplementary Figure S4a). Therefore, methylation of the alternative *BIM*-promoter does not correlate with the apoptosis-resistance-phenotype in NB4/FOXO3 and NB8/FOXO3 cells.

The deacetylation of FOXO3 by sirtuins has been described to significantly influence target-gene-regulation by FOXO3.^33, 34, 35^ However, the results in Supplementary Figure S4b did not reveal any differences in the acetylation-status of FOXO3, suggesting that overall acetylation/deacetylation of FOXO3 is not responsible for the observed phenotype.

#### The transcriptional activity of FOXO3 is steered by physical interaction with TP53

**Figure 4:**
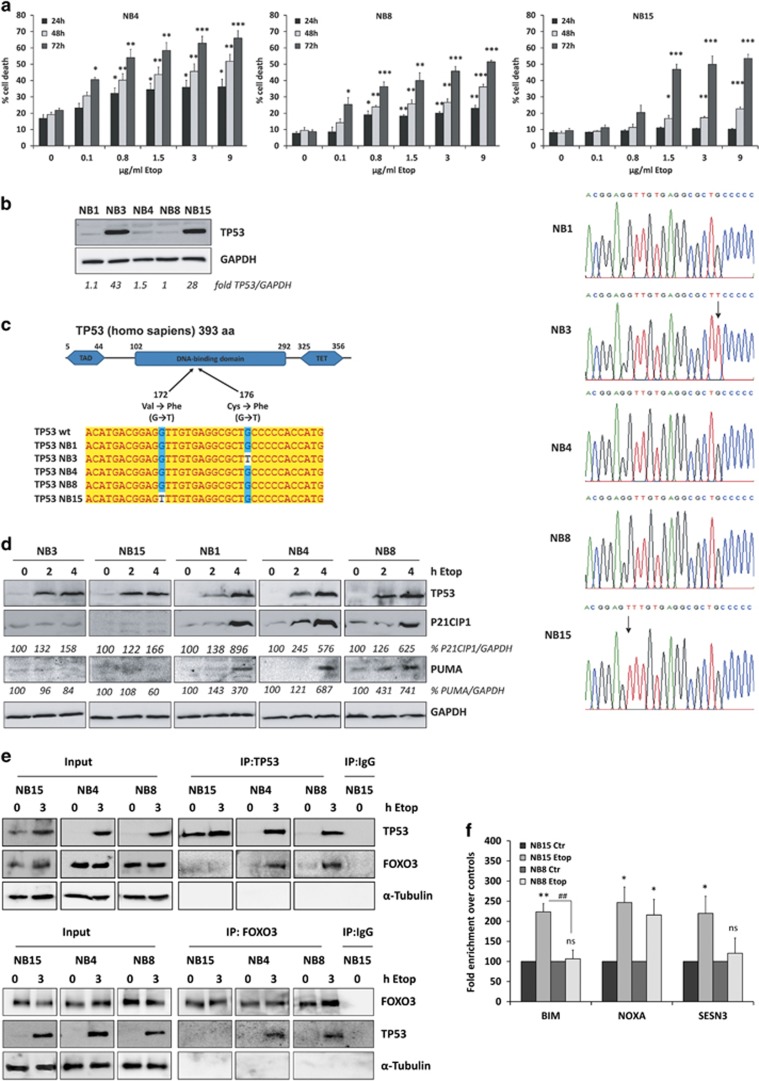
Binding of TP53 to FOXO3 affects target-gene-transcription in high-stage-derived NB cell lines. (**a**) NB4, NB8 and NB15 cells were treated with increasing doses of etoposide (0.1–9 μg/ml). Cell death was analyzed by PI-FACS analyses after 24, 48 and 72 h. Shown is the mean±s.e.m of three independent experiments; ***P<0.001, **P<0.01, *P<0.05; significantly different to untreated controls. (**b**) Cell lysates of NB1, NB3, NB4, NB8 and NB15 cells were subjected to immunoblot analyses for TP53. GAPDH served as loading control. For quantification, the TP53 level of NB8 cells was set as 1. (**c**) Sanger DNA sequencing of TP53 cDNA from NB1, NB3, NB4, NB8 and NB15 cells. Schematic structure of the TP53 protein (GenBank: NP_000537.3): TAD, transactivation domain; TET, tetramerization domain. Alignment of wild-type TP53 and sequencing data of TP53 cDNA prepared from NB1, NB3, NB4, NB8 and NB15 cells. (**d**) Cell lysates of NB3, NB15, NB1, NB4 and NB8 cells treated with 10 μg/ml etoposide for 0, 2 and 4 h were analyzed by immunoblot using antibodies against P21CIP1, PUMA and TP53. GAPDH served as loading control. To prevent saturation, a short exposure time for TP53 detection in NB3 and NB15 samples was chosen. Densitometry was performed using Labworks software version 4.5 (UVP, UK). (**e**) NB15, NB4 and NB8 cells were treated with 10 μg/ml etoposide for 0 and 3 h and subjected to immunoprecipitation using anti-TP53 (upper panel) or anti-FOXO3 (lower panel) as precipitating antibody and precipitates were analyzed by immunoblot using antibodies directed against TP53, FOXO3 and α-Tubulin. IgG (1 μg) was used as negative control. (**f**) ChIP analyses were performed in NB15 and NB8 cells treated with 10 μg/ml etoposide for 2 h. Binding of FOXO3 to the promoter regions of *BIM, NOXA* and *SESN3* were quantified by quantitative RT–PCR. Shown is the mean value±s.e.m. of three independent experiments, each performed in duplicate. Significantly different to untreated cells: **P<0.01, *P<0.05; significantly different between etoposide-treated cells: ^##^*P*<0.01. ChIP, chromatin-immunoprecipitation

Co-factor-binding can significantly modulate target-gene-regulation of transcription factors. At diagnosis, mutations of TP53 are rare and loss-of-function mutations are mostly detected in relapsed NB-tumors, which then correlate with increased chemotherapy-resistance.^21, 36^ Wild-type TP53 is usually hardly detectable due to the short half-life of this protein, but accumulates during DNA-damage. Elevated TP53 levels in the absence of genotoxic stress therefore suggest a mutation of the *TP53* gene.^37^ When treating NB cells with increasing concentrations of etoposide, NB4 and NB8 cells underwent cell death at lower doses than NB15 cells suggesting reduced sensitivity of NB15 cells to DNA-damaging agents (Figure 4a). By immunoblot analyses we observed different TP53-levels in high-stage NB cell lines. In FOXO3-resistant NB1, NB4 and NB8 cells TP53-expression was hardly detectable, whereas increased steady-state expression of TP53 was visible in NB3 and NB15 cells suggesting TP53-mutation (Figure 4b). Consequently, we sequenced the entire coding-region of TP53 and discovered that NB3 and NB15 cells carry homozygous mutations in the DBD of TP53. The G→T mutations at codon 172 (Val>Phe) in NB15 cells and at codon 176 (Cys>Phe) in NB3 cells flank the structural hotspot mutation R175H frequently found in advanced cancer^38^ (Figure 4c). The R175H mutation affects the TP53-conformation and hampers the TP53/ATM DNA-damage response. To test, whether the mutations found in NB3 and NB15 cells alter target-gene-induction by TP53, we induced DNA-damage-response by etoposide-treatment. In both subtypes, TP53 still significantly accumulated after etoposide-treatment: in NB1, NB4 and NB8 cells a three-to-nine-fold induction of the TP53 *bona fide* targets CDKN1A/P21CIP1 and BBC3/PUMA was observed, which indicates TP53-transcriptional function,^39^ whereas in NB3 and NB15 cells P21CIP1 was marginally induced and PUMA was not induced at all (Figure 4d). This suggests that the point-mutations in the DNA-binding-domain of TP53 in NB3 and NB15 cells impair transcriptional-activation of TP53 target-genes. Sequencing also revealed a TP53-base-exchange at codon 72 (C→G) in the linker region between the transactivating domain and the DBD in NB4 (heterozygous), NB1, NB8 and NB15 cells (homozygous) that was not seen in NB3 cells (Supplementary Figure S5). This represents a polymorphism which was described to correlate with increased risk for certain forms of cancer^40^—the relevance for NB development is not clear.

A physical interaction of the CR3-region and the forkhead (FH) domain of FOXO3 with the TP53-DBD that affects the activity of both transcription factors has been demonstrated before.^41, 42^ To investigate, whether the identified mutations in the TP53-coding-region differentially affect FOXO3–TP53-interaction in NB, we performed co-immunoprecipitation experiments. FOXO3 binds to TP53 in NB4 and NB8 cells after etoposide-treatment, but not in NB15 cells (Figure 4e). This suggests that the V172F-mutation in NB15 cells affects hetero-dimerization with FOXO3 and that FOXO3–TP53-complexes in NB4 and NB8 cells might interfere with distinct target-promoter-binding. To proof this hypothesis, we performed chromatin-immunoprecipitation-analyses on FOXO3-binding to the target-promoters *BIM*, *NOXA* and *SESN3* in response to etoposide-treatment. We observed increased FOXO3-binding to the *BIM*-promoter in NB15 cells after 2 h of etoposide-treatment, which was not visible in NB8 cells (Figure 4f), correlating with the results of the immunoblot analyses in Figure 3b. A slightly increased binding to the *SESN3*-promoter was seen in NB8 cells, whereas binding of FOXO3 to the *NOXA*-promoter showed similar increase by etoposide-treatment in both cell lines (Figure 4f). These results reflect the FOXO3-target-promoter-interaction previously observed with the FOXO3(A3)ERtm allele (Figure 2c). Also mRNA-regulation of these genes in response to etoposide-treatment showed induction of all three target genes only in NB15 cells, whereas in NB4 and NB8 cells only NOXA-levels increased (Supplementary Figure S6). Taken together, these data imply that under genotoxic stress FOXO3 forms a complex with TP53 in NB4 and NB8 cells, which prevents FOXO3-binding to the *BIM*-promoter and induction of BIM-protein, whereas the *NOXA*-promoter contains a TP53 binding site and hence FOXO3 may be recruited via TP53 to this promoter and sequestered away from the *SESN3*-promoter. In NB15 cells, however, the specific mutation of TP53 alters the interaction between FOXO3 and TP53 (Figure 4d), so that non-sequestered FOXO3 binds to the *BIM-*promoter (Figure 4f) and induces BIM-expression in response to etoposide-treatment (Figure 3b, Supplementary Figure S6).

### FOXO3-induced drug-protection depends on functional TP53 and SESN3

**Figure 5:**
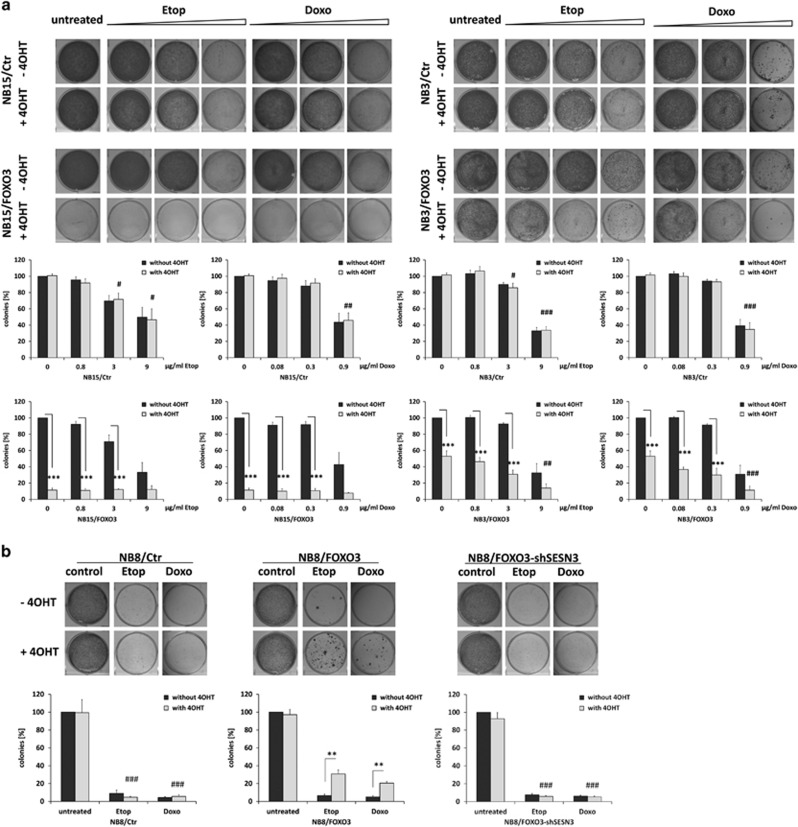

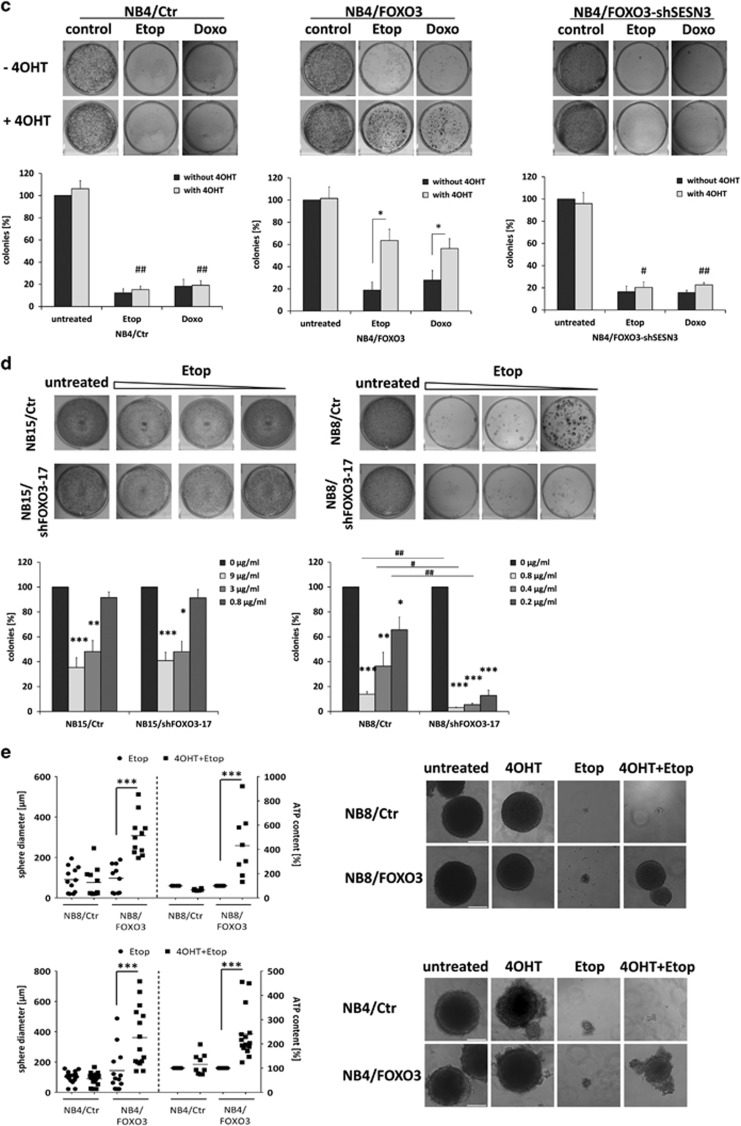
FOXO3 increases the resistance to chemotherapeutic agents in apoptosis-resistant cell lines. (**a**) Representative images of colony-formation-assays with NB15/Ctr, NB15/FOXO3 (left panel), and NB3/Ctr and NB3/FOXO3 cells (right panel) treated with 50 nM 4OHT for 72 h prior to combined treatment with 0.8/3/9 μg/ml etoposide or 0.08/0.3/0.9 μg/ml doxorubicin for another 72 h are shown. (**b**) Representative images of colony-formation-assays with NB8/Ctr, NB8/FOXO3 and NB8/FOXO3-shSESN3 cells treated with 50 nM 4OHT for 72 h prior to combined treatment with 0.8 μg/ml etoposide or 0.08 μg/ml doxorubicin for another 72 h are shown. (**c**) Representative images of colony- formation of NB4/Ctr, NB4/FOXO3 and NB4/FOXO3-shSESN3 cells treated with 50 nM 4OHT for 72 h prior to combined treatment with 0.1 μg/ml etoposide or 0.01 μg/ml doxorubicin for another 72 h. All colonies were stained with crystal violet and the amount of cells was quantified by photometry after discoloration with 0.5% SDS in 50% ethanol. Shown are means ±s.e.m. of four independent experiments; ***P<0.001, **P<0.01 *P<0.05 (between±4OHT within cell line) and ^###^*P*<0.001, ^##^*P*<0.01, ^#^*P*<0.05 (between 4OHT-treated cell lines). (**d**) Representative images of NB15/Ctr, NB15/shFOXO3-17, NB8/Ctr and NB8/shFOXO3-17 colony-formation-assays treated with 9 μg/ml, 3 μg/ml, 0.8 μg/ml (NB15 cells) or 0.8 μg/ml, 0.4 μg/ml and 0.2 μg/ml (NB8 cells) etoposide for 72 h. Colonies were stained with crystal violet. Quantification was performed by photometric measurement after discoloration with 0.5% SDS in 50% ethanol. Shown are means±s.e.m. of three independent experiments. ***P<0.001. **P<0.01 *P<0.05 (between untreated and etoposide treated), ^##^*P*<0.01, ^#^*P*<0.05 (between Ctr and shFOXO3 cells). (**e**) 3D spheroids of NB4/Ctr, NB4/FOXO3, NB8/Ctr and NB8/FOXO3 cells were formed by magnetic bioprinting for 72 h, treated with 50 nM 4OHT for 72 h prior to etoposide (0.1 μg/ml) treatment for another 72 h. Shown are sphere diameters and ATP values for each sphere from three independent experiment; Statistical differences (±4OHT) were assessed by Mann–Whitney test ^###^*P*<0.001, ^##^*P*<0.01.

To investigate how TP53/FOXO3 complexes in NB4 and NB8 cells affect the response to chemotherapy we performed colony-formation-assays to determine clonogenic-survival of these cells after exposure to chemotherapeutic agents. Ectopic FOXO3-activation in the FOXO3-responsive NB3 and NB15 cells leads to less colony-formation compared to control cells, but in presence of chemotherapeutics higher doses of etoposide and doxorubicin were needed to significantly reduce colony-formation capacity in these cells (Figure 5a). As shown in Figures 5b and c and Supplementary Figure S7, activation of FOXO3 significantly enhanced clonogenic-survival of NB8/FOXO3, NB4/FOXO3 and of NB1/FOXO3 cells compared to controls during etoposide- and doxorubicin-treatment. Ectopic expression of 4OHT-regulated FOXO3 *per se* slightly increases clonogenic-survival in these cells, which might result from slight leakiness of the construct, as described before.^14^ Also, endogenous FOXO3 exerts a pro-survival effect in this subtype, as FOXO3-knockdown (Supplementary Figure S8c)^3^ was able to increase drug-sensitivity only in NB8 cells, but not in NB15 cells (Figure 5d). The pro-survival phenotype of FOXO3 was also visible in 3D-tumor-spheroids, where FOXO3-activation significantly increases spheroid-size and viability as measured by ATP-content (Figure 5e).

Since ectopic FOXO3 binds to the *SESN3*-promoter and induces its transcription (Figures 2b and c) we studied whether the FOXO3-mediated pro-survival phenotype during chemotherapy might involve SESN3. Therefore, we knocked-down SESN3 by shRNA and analyzed colony-formation. Knockdown of SESN3 was verified by qRT–PCR (Supplementary Figure S8a) and significantly reduced the chemo-protective effect of FOXO3 in NB8/FOXO3-shSESN3 cells (Figure 5b). Consistent with the slightly less efficient knockdown in NB4/FOXO3-shSESN3 cells drug-resistance was also reduced to a lesser extent in this second cell line and increased basal resistance of FOXO3-transgenic cells was completely abrogated (Figure 5c). Drug-treatment in these SESN3-knockdown cells also led to significantly increased ROS-accumulation (Supplementary Figure S8b), suggesting that ROS-induction by FOXO3 might be re-activated in these cells. This implies that SESN3 contributes to FOXO3-mediated drug-protection in NB4 and NB8 cells.

**Figure 6:**
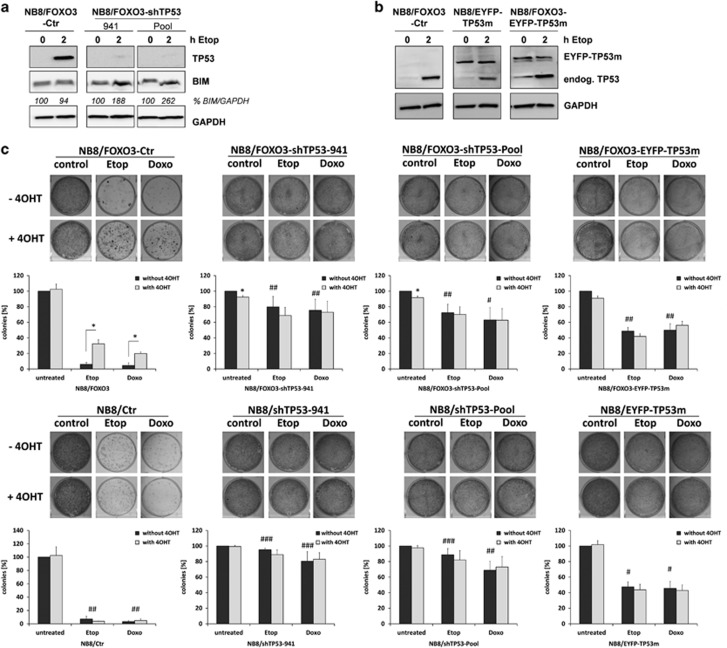

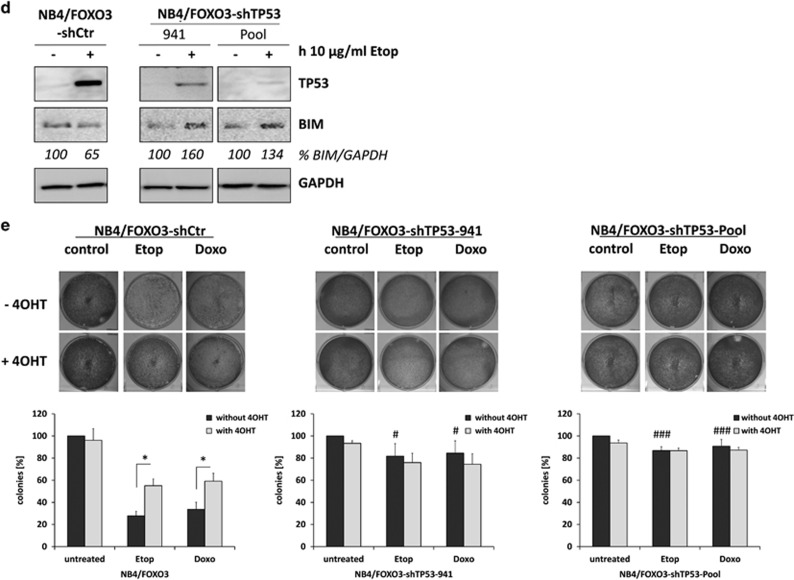
TP53 inhibition abolishes FOXO3-mediated chemo-protection. (**a**) Immunoblot analyses of TP53 and BIM in NB8/FOXO3-Ctr and TP53-knockdown cells as indicated. GAPDH served as loading control. Densitometry was performed using Labworks software version 4.5 (UVP, UK). (**b**) NB8/FOXO3-Ctr, NB8/EYFP-TP53m and NB8/FOXO3-EYFP-TP53m cells were analyzed for TP53 and BIM expression by immunoblot after treatment with 10 μg/ml etoposide for 2 h. (**c**) NB8/Ctr, NB8/FOXO3-Ctr and TP53 knockdown cells or NB8/EYFP-p53m and NB8/FOXO3-EYFP-p53m cells were treated for 72 h with 50 nM 4OHT before 0.8 μg/ml etoposide or 0.08 μg/ml doxorubicin were added for another 72 h. Colonies were stained with crystal violet. Quantification was performed by photometric measurement after discoloration with 0.5% SDS in 50% ethanol. Shown are means±s.e.m. of three independent experiments; *P<0.05 (between±4OHT within cell line) and ^##^*P*<0.01, ^#^*P*<0.05 (between drug-treated cell lines). (**d**) Immunoblot analyses of TP53 and BIM in NB4/FOXO3-shCtr and TP53 knockdown cells as indicated. GAPDH served as loading control. Densitometry was performed using Labworks software version 4.5 (UVP, UK). (**e**) NB4/FOXO3-shCtr and -TP53 knockdown cells were treated for 72 h with 50 nM 4OHT before 0.1 μg/ml etoposide or 0.01 μg/ml doxorubicin were added for another 72 h. Colonies were stained with crystal violet. Quantification was performed by photometric measurement after discoloration with 0.5% SDS in 50% ethanol. Shown are means±s.e.m. of three independent experiments; *P<0.05 (between±4OHT within cell line) and ^##^*P*<0.01, ^#^*P*<0.05 (between drug-treated cell lines).

To determine whether TP53-FOXO3 interaction is critical for the lack of BIM-induction, we repressed TP53 by short-hairpin RNA against TP53 and verified the knockdown by immunoblot after 2 h of etoposide-treatment (Figures 6a and d). Interestingly, the knockdown of TP53 partially restored etoposide-triggered BIM-induction and abrogated the FOXO3-imposed drug-protection-phenotype in NB8/FOXO3 and in NB4/FOXO3 cells. The efficient knockdown of functional TP53 was reflected by the increased basal resistance to chemotherapeutic drugs similar to NB15 cells (Figures 6c and e). Finally, we amplified the mutated TP53-coding-sequence from NB15-cDNA and cloned it downstream of EYFP into a retroviral vector. After infection of NB8 and NB8/FOXO3 cells the expression of the EYFP-TP53mut-fusion-protein was verified by immunoblot (Figure 6b). As for TP53-knockdown, expression of TP53-V172F-protein increased basal drug-resistance, but impaired the FOXO3-imposed pro-survival phenotype (Figure 6c), proving that the disruption of FOXO3–TP53-complexes by this mutation abrogates the FOXO3-induced drug-protection in NB cells.

## Discussion

Tumor promotion by FOXO3 has been discussed for several years.^17, 19, 43, 44, 45, 46^ Here, we identified a subtype of NB cells, where FOXO3-activation protected the cancer cells against DNA-damaging chemotherapeutic agents. The drug-resistance was mainly regulated by the interaction between wild-type TP53 and FOXO3, which alters FOXO3-promoter-recognition. Hyper-activation of the phosphatidylinositol3-kinase/PKB signaling pathway frequently observed in NB is associated with FOXO3-inactivation and its re-localization into the cytoplasm.^4, 5, 11, 12, 13^ However, in high-stage-derived NB cell lines FOXO3 partly localizes to the nucleus despite high PKB-activity and regulates the response to hypoxia within these cells (Figure 1).^14^ Activation by etoposide-treatment further increases the amount of nuclear FOXO3 (Figure 1c) suggesting that shuttling and oxidative stress response regulation of FOXO3 is still intact within these cells. Whereas ectopic activation of FOXO3 efficiently induces cell-cycle-arrest and apoptosis in NB15/FOXO3 and NB3/FOXO3 cells,^3, 5^ NB1/FOXO3, NB4/FOXO3 and NB8/FOXO3 cells remained insensitive to FOXO3 (Figure 1e and Supplementary Figure S2). These observations demonstrate that nuclear FOXO3 does not cause resistance against FOXO3-induced apoptosis *per se,* but that additional factors may modulate the physiological outcome. Analyses of target-gene-regulation uncovered that the apoptosis-inducing gene BIM which is critical for death-execution after FOXO3-activation in NB cells^3, 5, 47^ was not induced in NB4/FOXO3 and NB8/FOXO3 cells. Other FOXO3-targets were still regulated in these cells, although in part to a lesser extent (Figures 2a and b and Supplementary Figure S3). The nuclear accumulation of FOXO3 and the induction of P27KIP1, however, demonstrate that the ectopic FOXO3(A3)ERtm construct was active in all cell lines. Since BIM is the key player in FOXO3-induced death that triggers ROS-accumulation,^3^ the lack of BIM-regulation might be crucial for absence of FOXO3-induced death. The transcriptional activity of FOXO3 is strongly affected by post-translational modifications. Deacetylation by sirtuins reprograms the activity of FOXO3 at oxidative stress conditions towards pro-survival target genes, like SOD2 and GADD45A instead of BIM and P27KIP1.^48^ However, we did not observe significant differences in the FOXO3-acetylation-status between resistant and sensitive cell lines (Supplementary Figure S4b). This most likely excludes acetylation/deacetylation of FOXO3 as a critical factor for BIM-expression. By chromatin-immunoprecipitation-analyses we uncovered that FOXO3 was not recruited to the *BIM*-promoter in resistant cell lines (Figure 2c). Since we did not detect any changes in methylation on the *BIM*-promoter in NB8/FOXO3 and NB4/FOXO3 compared to NB15/FOXO3 cells (Supplementary Figure S4a, Supplementary Table S4) and methylation-patterns of an alternative *BIM*-promoter did not correlate with the resistance-phenotype, we also excluded differential-methylation as the cause for lack of BIM-induction in NB4 and NB8 cells. The N-terminus of TP53 binds to the FH-domain of FOXO3 and in parallel the CR3-domain of FOXO3 interacts with the DBD of TP53,^41^ which alters target-recognition by FOXO3.^42^ Inhibition of one of these two interaction-interfaces, for example, of the TP53-DBD/FOXO3-CR3-region, prevents complex formation. We observed that TP53 and FOXO3 form complexes in the FOXO3-resistant NB-subtype (NB4 and NB8) upon exposure to etoposide, but not in the FOXO3-sensitive NB-subtype (NB15, Figure 4e), which might be explained by the TP53 mutations V172F (NB15) and C176F (NB3) (Figure 4c). These mutations reduce TP53-transcriptional-activity as demonstrated by reduced P21CIP1 and PUMA-induction after DNA-damage (Figure 4d) and prevent its interaction with FOXO3 (Figure 4e), whereas the polymorphism at codon 72 (C→G) (Supplementary Figure S5) did not have an effect (Figure 4d). Many inactivating mutations of TP53 have been described also in NB,^36^ but to our knowledge not for V172F. TP53-V172F was recently described as a temperature-sensitive TP53 mutant that affects cisplatin-sensitivity in ovarian cancer.^49^ However, V172F and C176F flank the structural hotspot mutation locus R175H in the Zn^2+^-binding region that changes TP53-conformation. R175H is the most common mutation found in human cancer that impairs DNA-binding and ATM-mediated DNA-damage-response, thereby leading to genetic instability and defective G2/M checkpoint control.^50^ Further analyses on how this TP53/FOXO3-complex affects cell-fate uncovered drug-protection by FOXO3-activation, whereas knockdown of endogenous FOXO3 increased drug-sensitivity (Figure 5). This is in line with several studies which already identified correlations between FOXO3 and drug-resistance in different cancer types.^17, 18, 19, 43, 44^ Detailed analyses of target-gene-regulation after FOXO3-activation by etoposide or direct activation of the FOXO3(A3)ERtm-protein uncovered that BIM is not induced in resistant cell lines, whereas NOXA was induced in both, FOXO3-resistant and -sensitive cell types (Figures 2, 3b and 4f). Although FOXO3 binds to the *SESN3*-promoter and induces SESN3 after 4OHT-activation, only partial increase in *SESN3*-promoter-binding or SESN3-induction were observed in chemo-resistant NB4 and NB8 cells after etoposide-treatment. One explanation is that during DNA-damage, endogenous FOXO3 is sequestered by accumulating functional TP53, thereby also preventing further binding to the *SESN3*-promoter. The *NOXA*-promoter in contrast contains TP53- as well as FOXO3-binding sites allowing the recruitment of the TP53/FOXO3-complex to this promoter. When elevated levels of active FOXO3 are present, as observed during hypoxia in growing NB tumors^14^ (or mimicked by ectopic FOXO3-ERtm) the *SESN3*-promoter is bound and FOXO3-activation increases cell survival/colony-formation during drug treatment.

In NB15/FOXO3 cells, however, SESN3 was induced by both ectopic FOXO3 and etoposide (Figures 2 and 4f). Since knockdown of SESN3 or TP53 abolished the chemo-protective function of FOXO3 (Figures 5b and c and Figure 6), our data suggest that NB1, NB4 and NB8 cells benefit from nuclear/active FOXO3 by already elevated SESN3-expression, which allows survival even under high-stress conditions after DNA-damage. We demonstrated before that SESN3 mediates ROS-detoxification in NB15 cells,^3^ but this protective effect is overcome by continuous mitochondrial damage via BIM, which eventually leads to death in these cells (Figure 1e).^3^ In FOXO3-resistant cells, however, endogenous TP53 forms a complex with FOXO3, which prevents BIM-induction.

**Figure 7:**
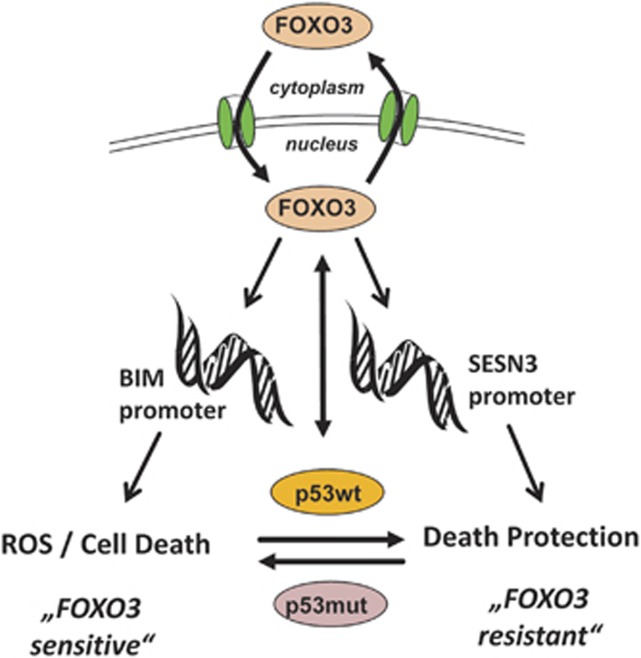
Proposed mechanism for the effect of functional TP53 on FOXO3-mediated drug-resistance in high-stage-derived NB. In the ‘FOXO3-sensitive’ subtype (NB15, NB3 cells) the mutation in the DBD of TP53 (p53mut) prevents interaction with FOXO3 during chemotherapeutic stress, so that FOXO3 steps in as tumor-suppressor and induces BIM to trigger death. This results in BIM and SESN3 induction and the pro-survival effect of SESN3 is eventually overcome by continuous accumulation of mitochondrial ROS. In the ‘FOXO3-resistant’ subtype (NB1, NB4, NB8), death induction via BIM is prevented by the interaction of TP53 with FOXO3. In these cells, TP53 serves as death-inducer in response to drug-treatment leading to lower basal drug resistance—but as soon as FOXO3 is activated, for example, by hypoxia or other stress signals in NB tumors, FOXO3 increases drug-protection. This mechanism might also explain the low incidence of TP53 mutations in malignant NB, as the TP53-FOXO3 interaction forces FOXO3-induced detoxification and prevents FOXO3-induced death.

In summary, our data describe a pro-survival function of FOXO3 in high-stage NB which depends on distinct target-gene-regulation under low- and high-stress conditions (Figure 7). In NB, TP53-mutations are rare and mainly detected in biopsies from relapsed patients. Our study uncovered that wild-type TP53 changes the target-gene-regulation of FOXO3 and thereby contributes to death protection. Targeting the transcriptional activity of FOXO3 and/or the interfaces between TP53 and FOXO3 might therefore be a promising approach for the treatment of therapy-resistant NB patients with functional TP53.

## Materials and methods

### Cell lines and reagents

The NB cell lines STA-NB1, STA-NB3, STA-NB4, STA-NB8 and STA-NB15, provided by St. Anna children’s Hospital, Vienna, are termed NB1, NB3, NB4, NB8 and NB15, respectively. Phoenix packaging cells for the production of retroviruses^51^ and NB cells were cultured in RPMI1640 medium (Lonza, Basel, Switzerland) supplemented with 10% fetal bovine serum (Sigma-Aldrich, St Louis, IL, USA), 100 U/ml penicillin, 100 μg/ml streptomycin and 2 mM L-glutamine (Lonza) in 5% CO_2_, 95% relative humidity and 37 °C. HEK293T cells for the lentivirus-production were cultured in Dulbecco’s modified Eagle’s medium (Invitrogen, Carlsbad, CA, USA). All cell lines were routinely tested for mycoplasma-contamination using the Venor GeM-Mycoplasma-detection-kit (Minerva Biolabs, Berlin, Germany).

### Retroviral and lentiviral expression vectors

The retroviral vectors pLIB-MCS2-iresNeo and pLIB-FOXO3(A3)-ERtm-iresNeo have been described.^5^ The lentivirus-vector coding for FOXO3-specific shRNA (pLKO-shFOXO3-91617-puro) and SESN3-specific shRNA (pLKO-shSESN3-141228-puro and pLKO-shSESN3-143446-puro) and the control-vector pLKO.1 were described.^3^ The lentivirus-vectors coding for TP53-specific shRNA pLKO-shTP53-puro, pLKO-shTP53-941-puro and pLKO-shTP53-427-puro were provided by T. Waldmann,^52^ the pGL3-Bim-promoter was provided by A. Villunger.^53^ The vector pLIB-EYFP-TP53mut-puro was generated by amplification of mutant-TP53 from NB15 cDNA (p53-fwd: 5′-GAATTCATGGATGATTTGATGC-3′ p53-rev: 5′-GGATCCTCAGTCTGAGTCAG)-3′ and inserted into the *Eco*R1- and *Bam*H1-site of pLIB-EYFP-Mcl1JAM-iresPuro^54^ replacing MCL1JAM by the TP53-mutant and generating pLIB-EYFP-p53m-iresPuro.

### Retroviruses and lentiviruses for infection

Retroviruses and lentiviruses were produced as previously described.^3^ pLIB-FOXO3(A3)-ERtm-iresNeo retrovirus-supernatants were used to infect NB4, NB8 and NB15 (NB4/FOXO3, NB8/FOXO3 and NB15/FOXO3). pLKO-shFOXO3-91617, pLKO-shSESN3-141228, pLKO-shSESN3-143446, pLKO-shTP53, pLKO-shTP53-941 and pLKO-shTP53-427 lentivirus-supernatants were used to infect NB8, NB8/FOXO3 and NB4/FOXO3 cells (NB8/shFOXO3-17, NB8/FOXO3-shSESN3, NB4/FOXO3-shSESN3, NB8/FOXO3-shTP53-941 and -Pool; NB4/FOXO3-shTP53-941 and -Pool). The vector pLIB-EYFP-p53mut-iresPuro was used to infect NB8 and NB8/FOXO3 cells (NB8/EYFP-TP53m, NB8/FOXO3-EYFP-TP53m). The empty vectors pLIB-MCS2-iresNeo and pLKO.1 served as controls (NB4/Ctr, NB8/Ctr, NB15/Ctr, NB8/shCtr, NB4/FOXO-shCtr, NB8/FOXO3-Ctr and NB15/shCtr).

### Flow cytometry

Cell death was determined by staining the cells with propidium iodide/Triton-X100 (Sigma-Aldrich) and forward/sideward-scatter-analysis using a Beckman Coulter Cytomics FC-500 as described previously.^55^

### Immunoblotting and co-immunoprecipitation

Preparation of total protein, cytoplasmic and nuclear fractions was described before.^3, 14^ Co-immunoprecipitation was performed as described^54, 56^ using 1 μg of TP53-antibody, FOXO3-antibody or control-IgG and 0.5% sodium-deoxycholate for effective nuclear lysis. Immunoblotting was done as described before.^3^ Primary antibodies (Supplementary Table S1) were detected with horseradish-peroxidase-conjugated secondary antibodies and chemiluminescence (GE Healthcare, Little Chalfont, UK) and analyzed using an UVP AutoChemi-detection-system.

### Chromatin-immunoprecipitation

Chromatin-immunoprecipitation was performed using Magna-ChIP-Kit (Millipore, Darmstadt, Germany) as described.^6^ protein-G-magnetic-beads (20 μl) were coupled to 7.5 μl of FOXO3 antibody (Santa Cruz Biotechnology, Dallas, TX, USA) or control-IgG and incubated with nuclear lysates of shredded DNA from 2 × 10^7^ cells. After precipitation, protein was digested by proteinase-K and DNA was concentrated with ChIP-DNA-Clean-&-Concentrator-Kit (Zymo Research, Irvine, CA, USA). FOXO3-binding to DNA was quantified by qPCR using promoter-specific primers for FOXO3-targets (Supplementary Table S2).

### Immunofluorescence and live cell imaging

Cells were seeded in eight-well μ-slides with glass-bottom (ibidi, Germany) coated with 0.1 mg/ml collagen. For immunofluorescence, cells were fixed with 4% ROTI-Histofix (Carl Roth, Karlsruhe, Germany) and permeabilized with 1% TritonX-100. After blocking with 1% bovine serum albumin cells were incubated with FOXO3-antibody (Cell Signaling, Boston, MA, USA), washed and incubated with anti-rabbit-AlexaFluor488 FITC-conjugated secondary antibody (Invitrogen). Nuclei were stained with 100 nM Hoechst33342 dye. For ROS-measurements, cells were incubated with CM-H_2_XROS (final concentration 500 nM; Invitrogen). Images were acquired with a Zeiss Axiovert200M microscope equipped with an ApoTome.2 for structured illumination microscopy.

### Quantitative RTPCR

To quantify mRNA-levels real-time qPCR was performed using Maxima-SYBR-Green-qPCR-Master-Mix (Thermo Scientific, Waltham, MA, USA) and GAPDH as reference-gene. Total-RNA was isolated from 5 × 10^6^ cells using TRI-Reagent (Sigma-Aldrich). RNA (1 μg) was transcribed to cDNA using RevertAid-H-Minus-cDNA-Synthesis Kit (Thermo Scientific). Oligonucleotides for BIM, NOXA, BCLXL, BIRC5, SESN3 and GAPDH are listed in Supplementary Table S3. qRT–PCR-reactions were performed in triplicates in a Bio-Rad-iCycler-instrument and repeated three times. After normalization to GAPDH, regulation was calculated between treated and untreated cells.

### TP53-sequencing

TP53 was amplified from cDNA using oligonucleotides listed in Supplementary Table S5. PCR-products were analyzed on 1% agarose gel and sequenced (Microsynth, Balgach, Switzerland).

### Colony-formation-assay

Cells were seeded in six-well-plates and treated with 4OHT for 72 h prior to the combined treatment with doxorubicin or etoposide for another 72 h. When untreated controls reached 100% confluence colonies were fixed with ice-cold methanol, stained with 0.2% crystal violet in 50% methanol and quantified by photometry at 600 nm after discoloration with 0.5% SDS in 50% ethanol.

### Spheroids

Spheroids were formed by ferromagnetic bioprinting according to the manufacturer’s instructions (Pelobiotech GmbH, Planegg, Germany). When spheroids reached a size of 100 μm the cells were treated with 4OHT for 72 h prior to the combined treatment with etoposide for another 72 h. Size was monitored regularly by live-cell microscopy. ATP-amount was measured by CellTiter-Glow-3D-cell-viability-assay (Promega, Madison, WI, USA).

### Statistics

Statistical significance of differences between controls and treated cells were assessed using Student’s unpaired *t*-test or with Mann–Whitney *U* test (for speroids). All statistical analyses and *P*-values were calculated using GraphPadPrism 7.0.
