# Supplementary material for: FOXO3-mediated chemo-protection in high-stage neuroblastoma depends on wild-type TP53 and SESN3
Source: Oncogene. 2017 Sep 4;36(44):6190–203. doi: 10.1038/onc.2017.288 (PMC5671944; doi:10.1038/onc.2017.288)

# Supplemental Figure S1:

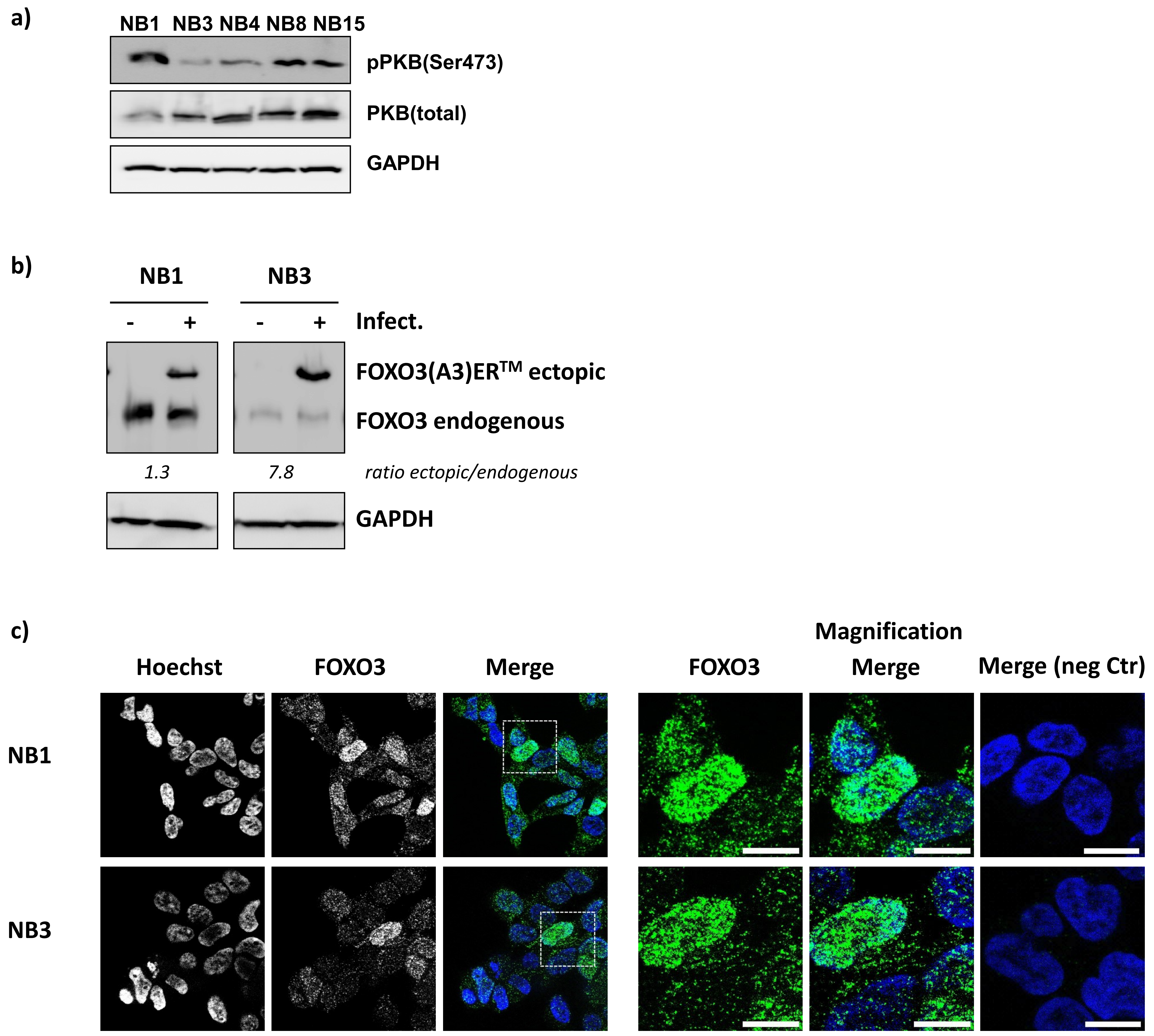

# Supplemental Figure S2:

**a)**

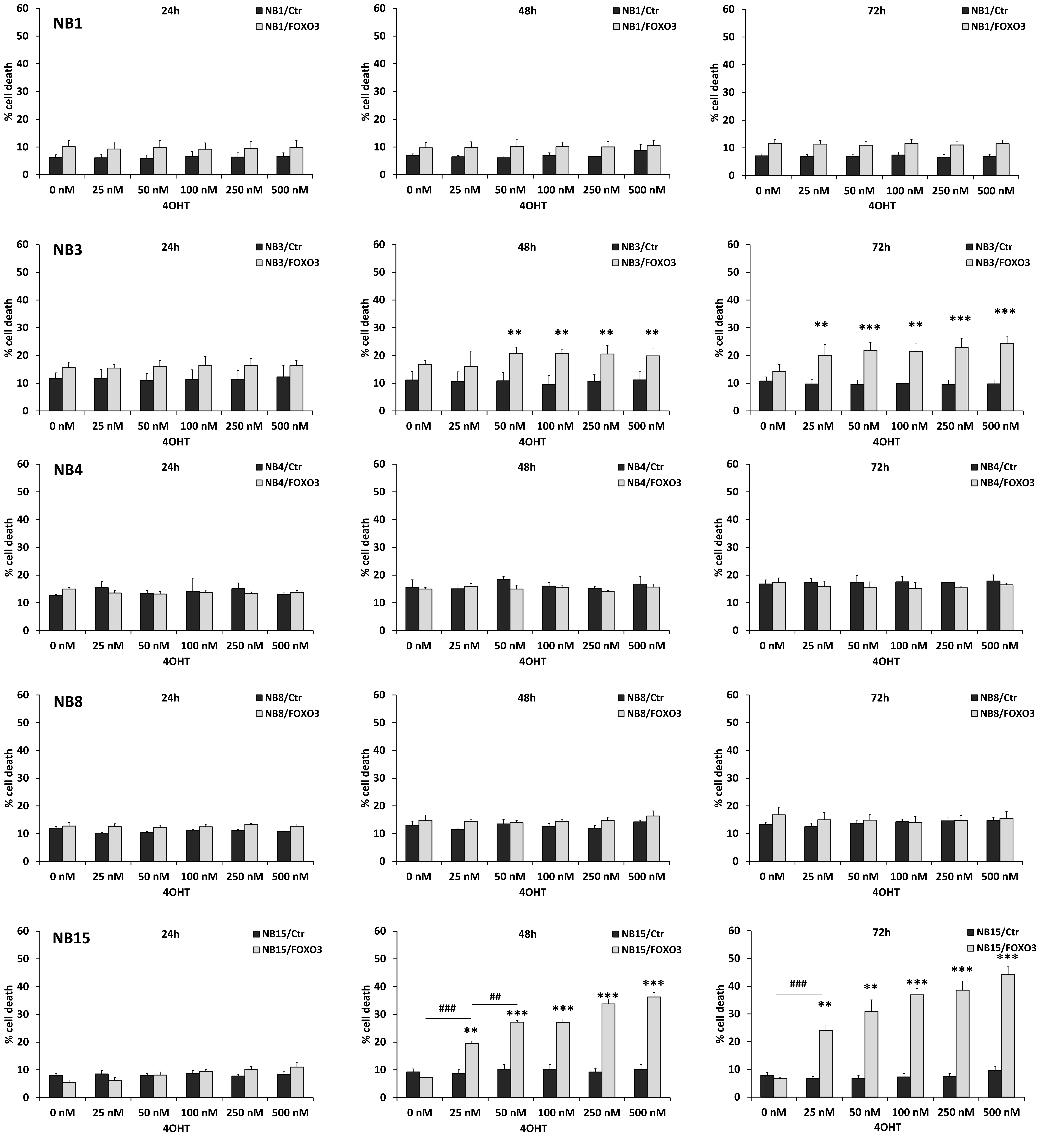

b)

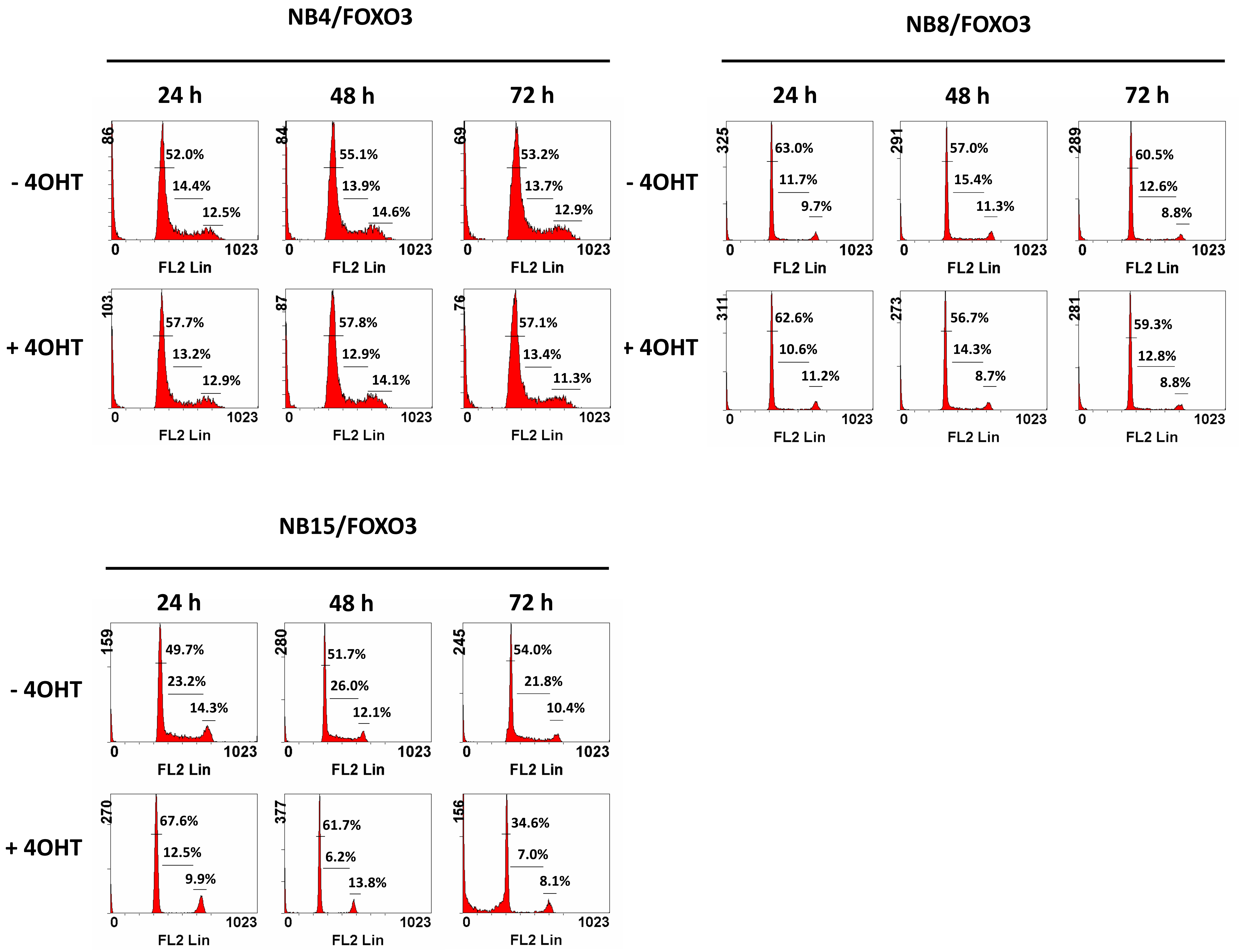
NB4/FOXO3
24 h
48 h
72 h
- 4OHT
+ 4OHT
86
52.0%
14.4%
12.5%
84
55.1%
13.9%
14.6%
69
53.2%
13.7%
12.9%
103
57.7%
13.2%
12.9%
87
57.8%
12.9%
14.1%
76
57.1%
13.4%
11.3%
0
1023
FL2 Lin
NB8/FOXO3
24 h
48 h
72 h
- 4OHT
+ 4OHT
325
63.0%
11.7%
9.7%
291
57.0%
15.4%
11.3%
289
60.5%
12.6%
8.8%
311
62.6%
10.6%
11.2%
273
56.7%
14.3%
8.7%
281
59.3%
12.8%
8.8%
NB15/FOXO3
24 h
48 h
72 h
- 4OHT
+ 4OHT
159
49.7%
23.2%
14.3%
280
51.7%
26.0%
12.1%
245
54.0%
21.8%
10.4%
270
67.6%
12.5%
9.9%
377
61.7%
6.2%
13.8%
156
34.6%
7.0%
8.1%

Supplemental Figure S3:

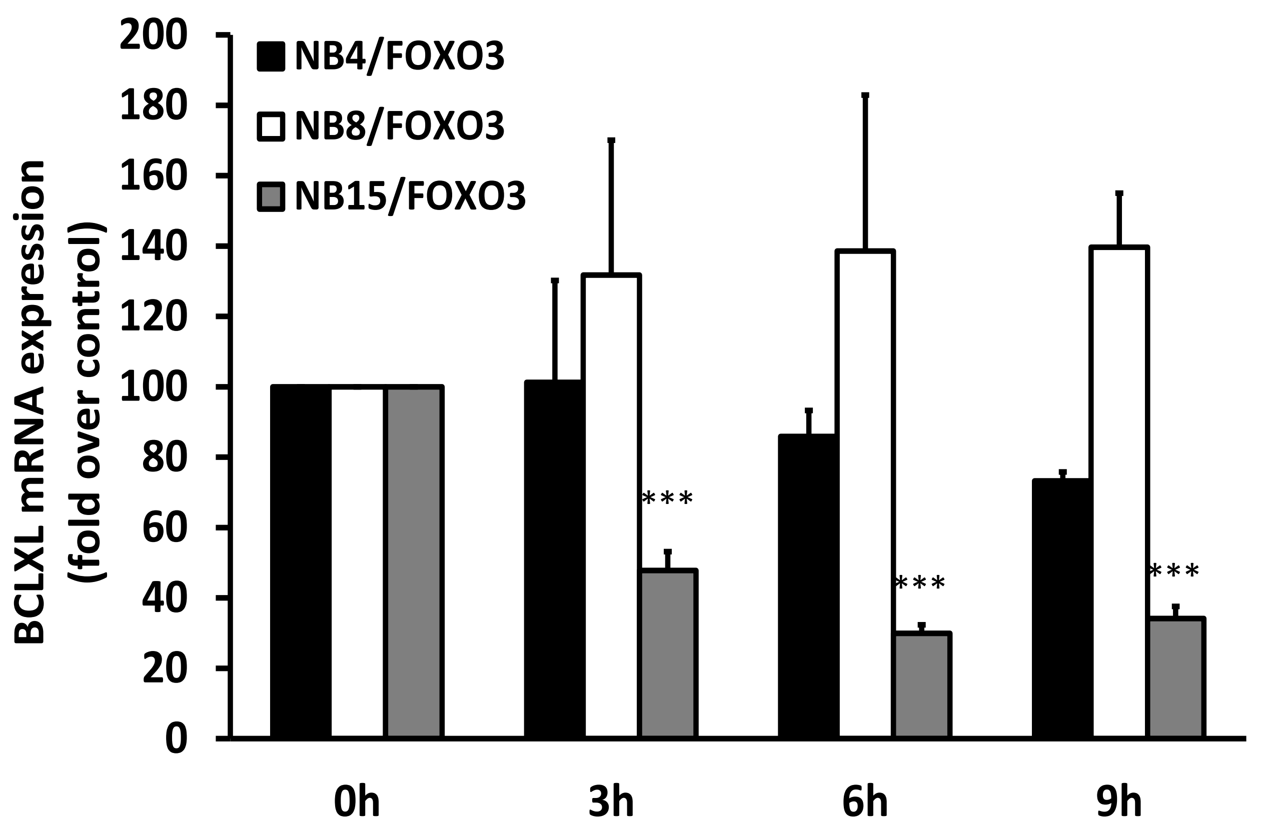


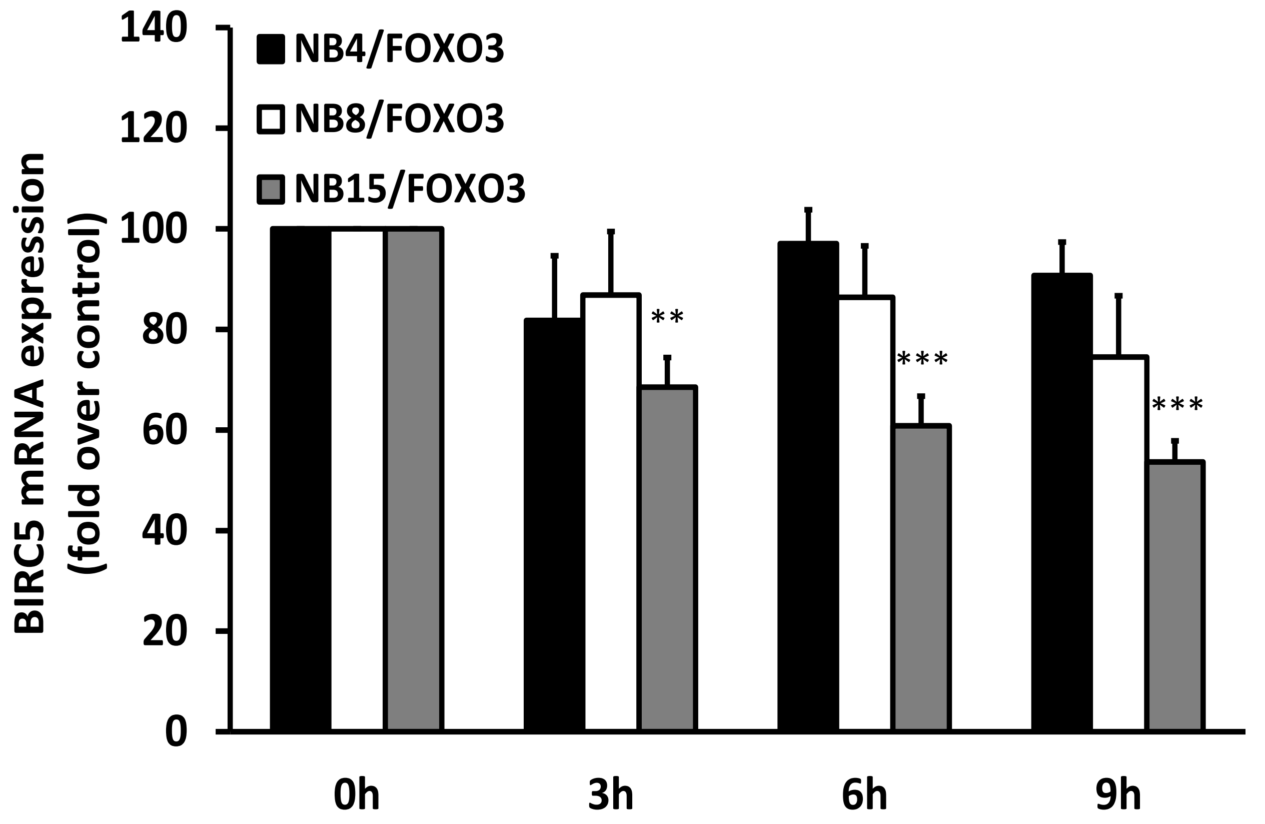

Supplemental Figure S4:

a)

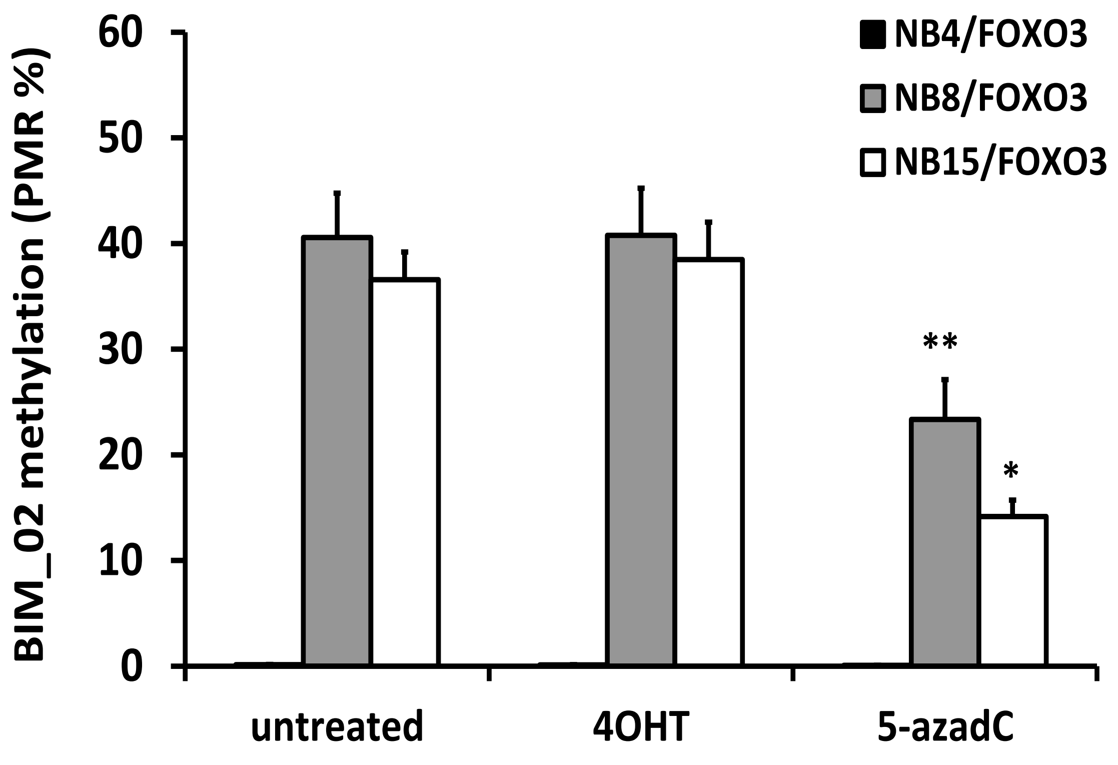


b)

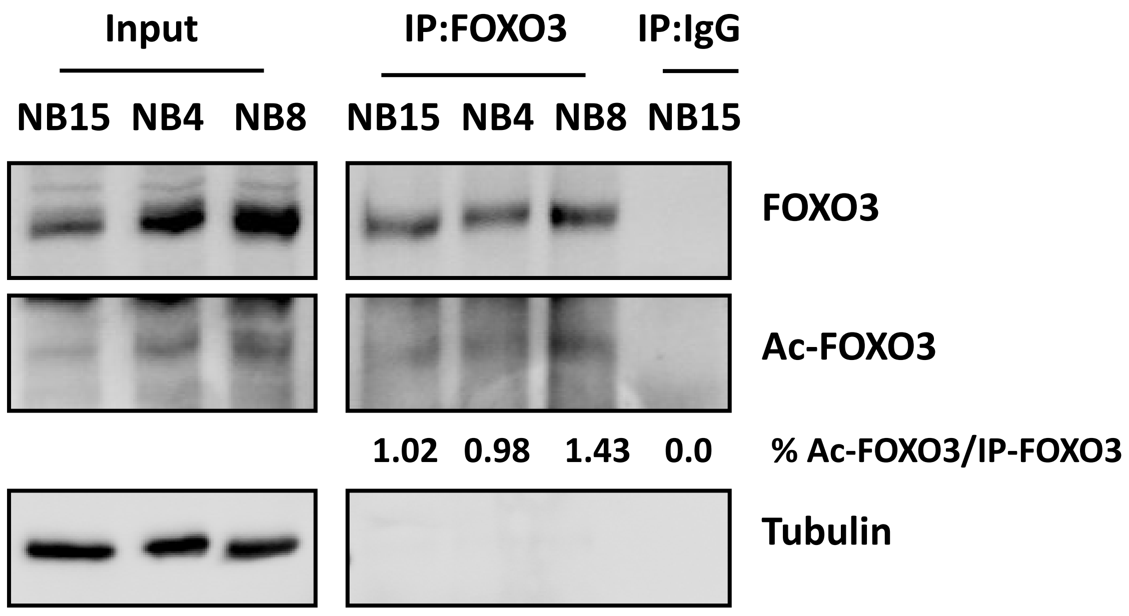

Supplemental Figure S5:

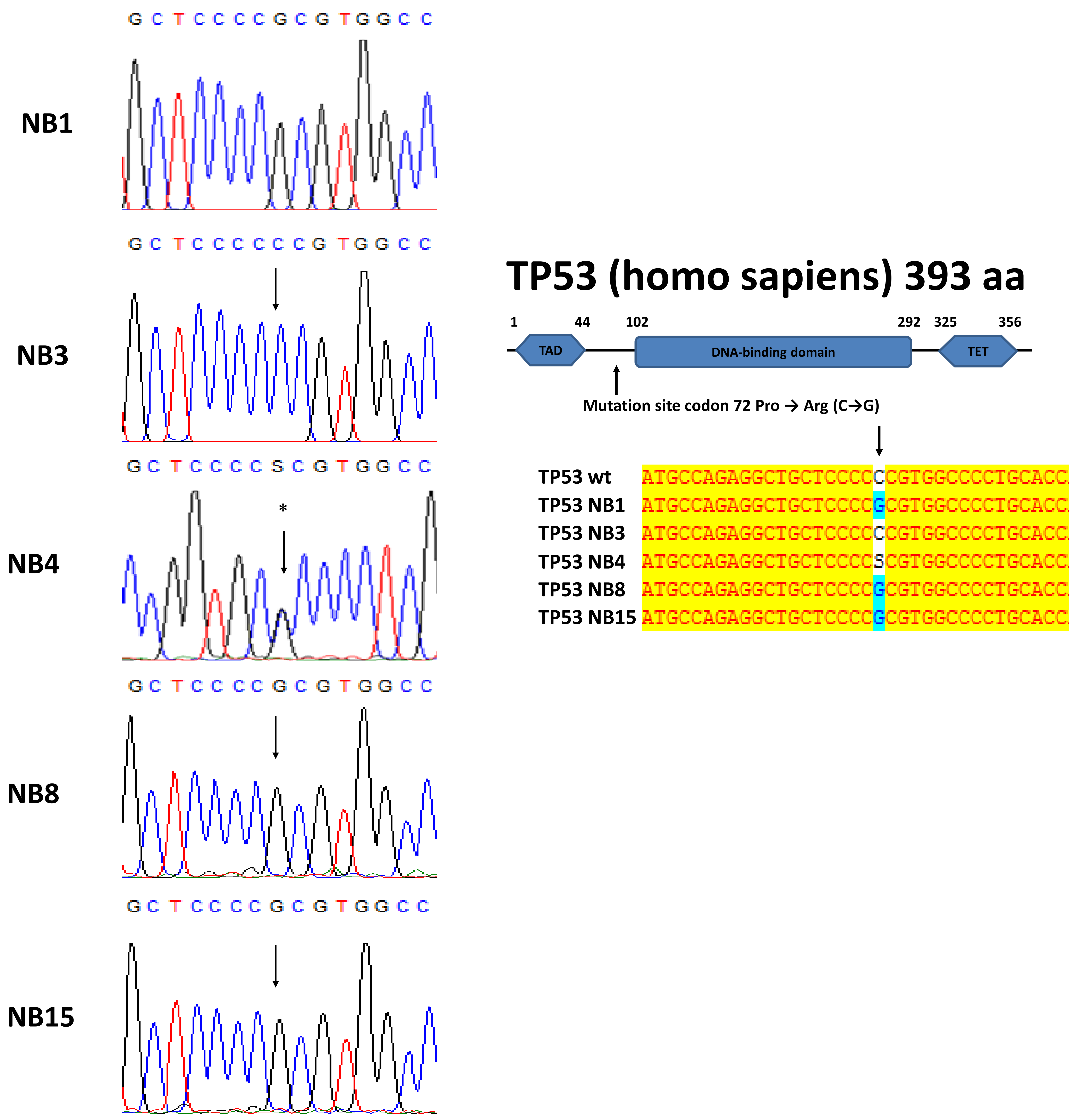

Supplemental Figure S6:

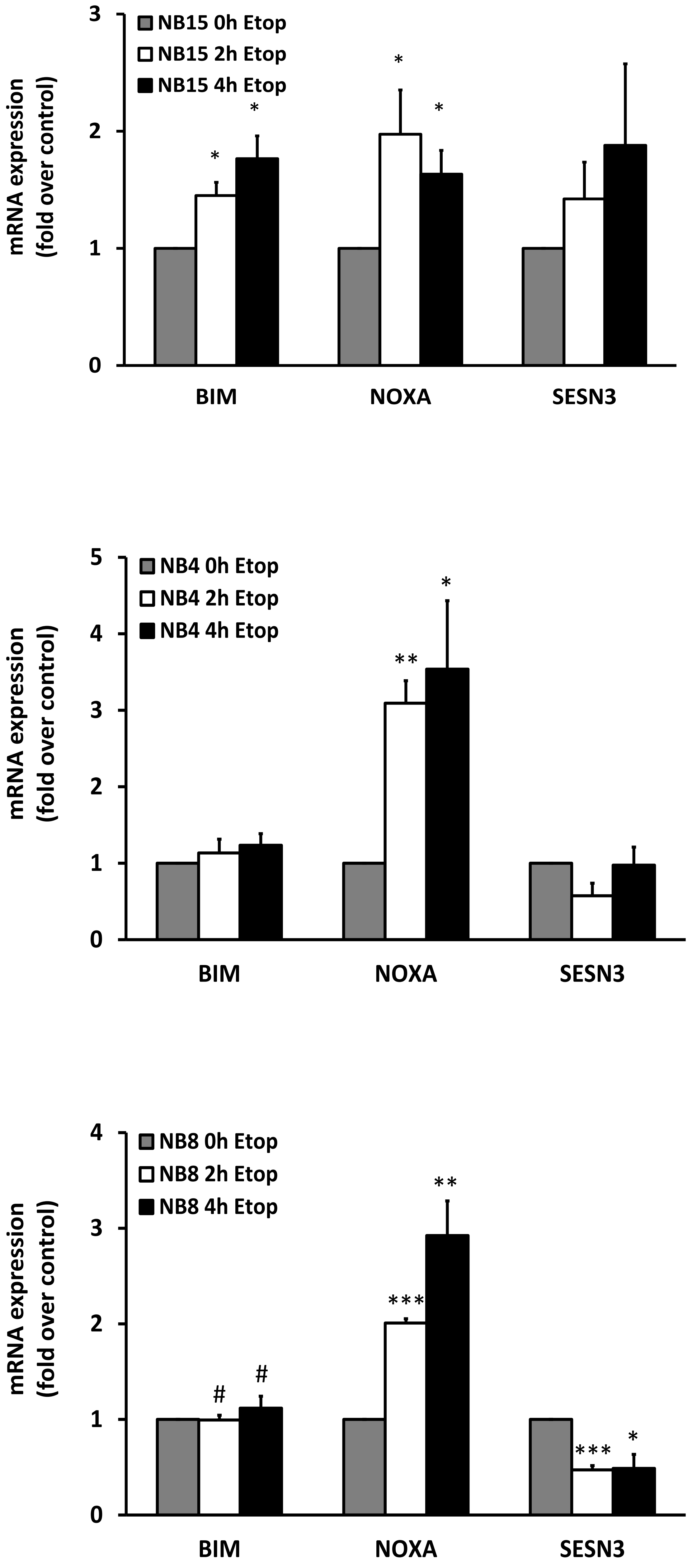

Supplemental Figure S7:

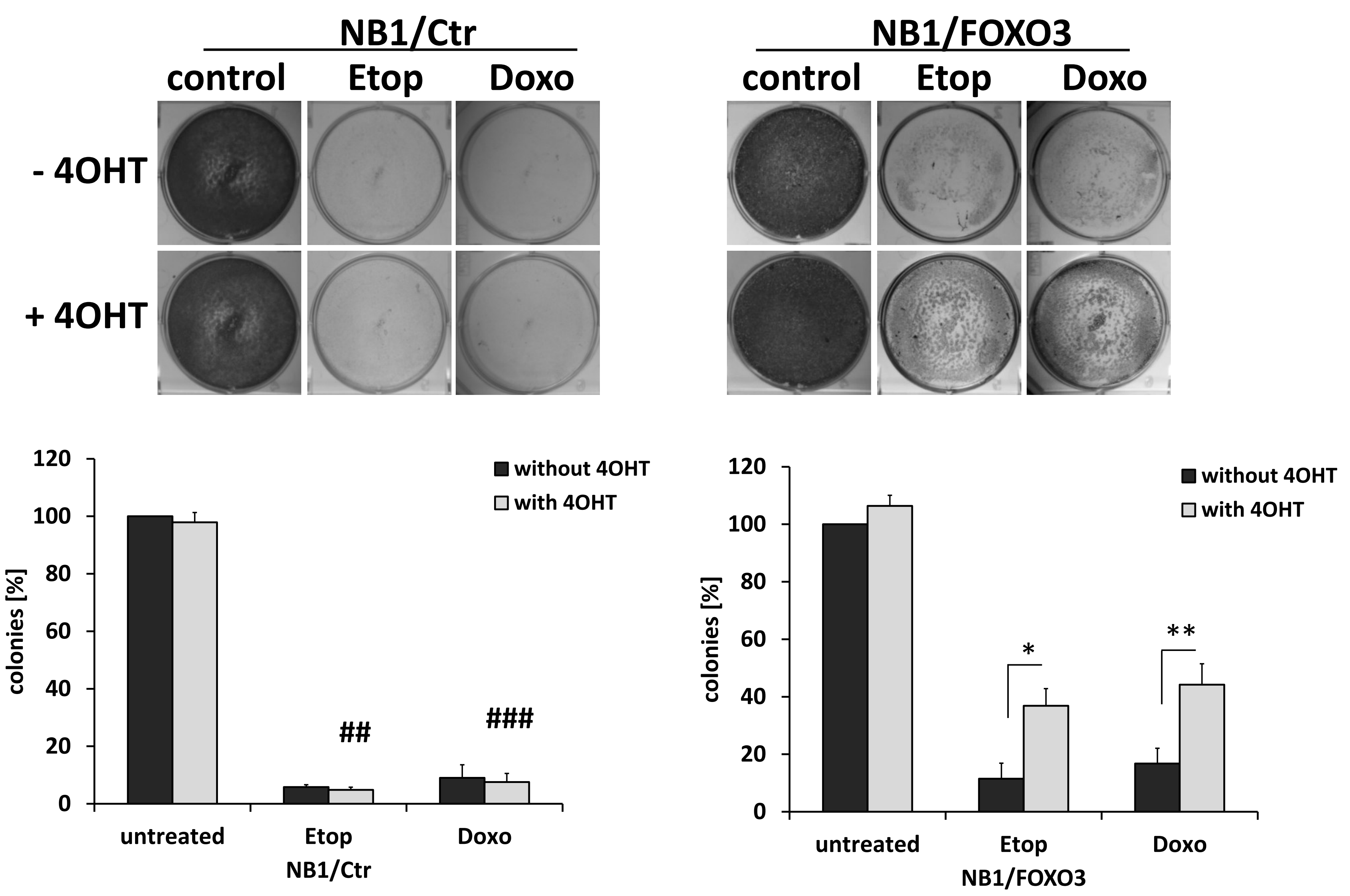

Supplemental Figure S8:

a)

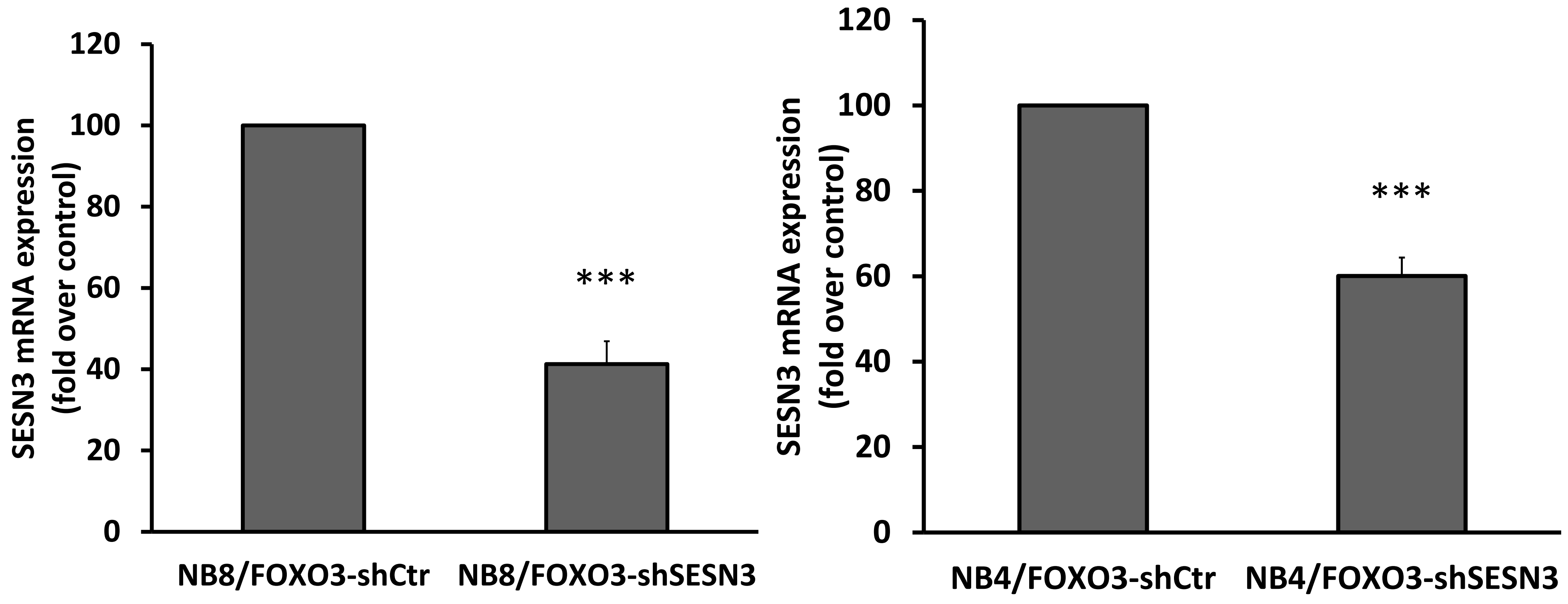


b)

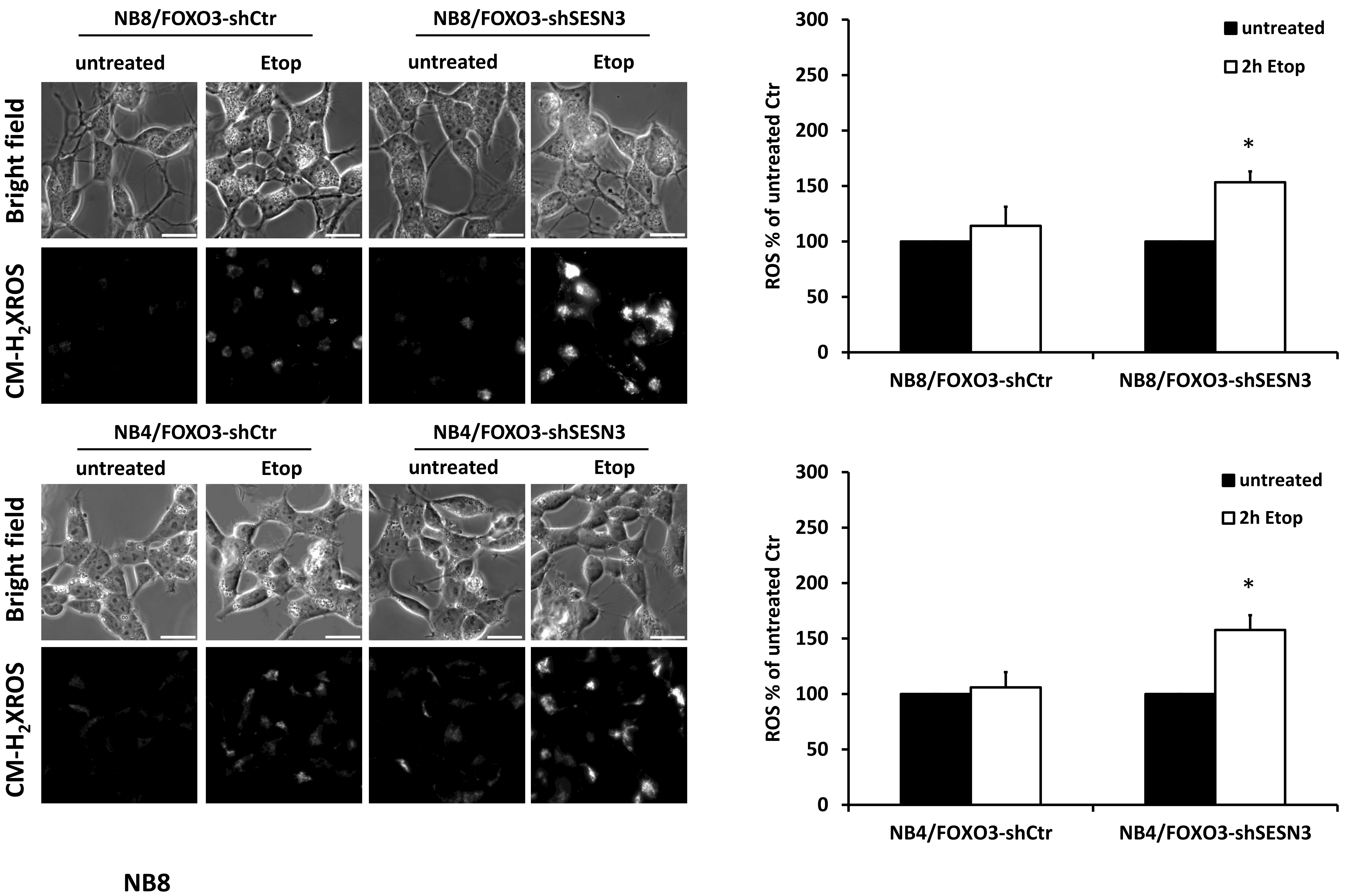


c)

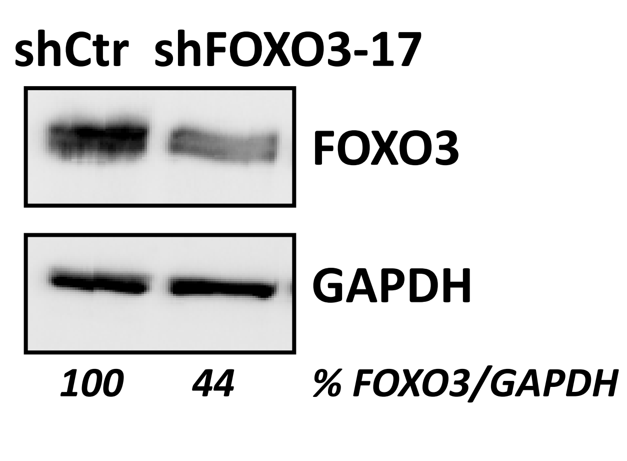

Supplement: Supplementary Figures [file onc2017288x1.pdf]
