# Supplementary material for: FOXO3-mediated chemo-protection in high-stage neuroblastoma depends on wild-type TP53 and SESN3
Source: Oncogene. 2017 Sep 4;36(44):6190–203. doi: 10.1038/onc.2017.288 (PMC5671944; doi:10.1038/onc.2017.288)
Supplement: Supplementary Information [file onc2017288x2.docx]

**Legends for Supplemental Figures**

**Supplemental Figure S1: Characterization of NB cell lines.** (a) The patient-derived NB cells NB1, NB3, NB4, NB8, and NB15 were analyzed for the expression and phospho-status of PKB and pPKB-S473 by immunoblot. GAPDH served as loading control. (b) Verification of transgene expression in NB1 and NB3 cells after retroviral infection with the pLIB-FOXO3(A3)ERtm-Neo plasmid. (c) NB1 and NB3 cells were fixed with 4% ROTI®-Histofix and stained with an antibody against FOXO3. Nuclei were visualized with Hoechst33342 dye. Images were acquired by an Axiovert200M microscope equipped with an ApoTome2. Bar is 10 µm.

**Supplemental Figure S2: Effect of ectopic FOXO3-activation.** (a) NB1/Ctr, NB1/FOXO3, NB3/Ctr, NB3/FOXO3, NB4/Ctr, NB4/FOXO3, NB8/Ctr, NB8/FOXO3, NB15/Ctr, and NB15/FOXO3 cells were treated with increasing concentrations of 4OHT (25-500 nM) for 24, 48, and 72 hours and subjected to cell death analyses by PI-FACS analyses. Shown are representative images and means ±s.e.m. of three independent experiments. ****P*<0.001. ***P*<0.01 (significantly different to control infected cell line); ###P<0.001, ##*P*<0.01 (significantly different between ascending 4OHT-concentrations) (b) Representative images of cell-cycle distribution in NB4/FOXO3, NB8/FOXO3, and NB15/FOXO3 cells. Cells were treated with 50 nM 4OHT for 24, 48, and 72 hours. Cell-cycle distribution was analyzed by flow cytometric analysis of PI-stained nuclei.^1^

**Supplemental Figure S3: Differential regulation of anti-apoptotic FOXO3 target genes.** (a) mRNA levels of BCLXL and BIRC5/survivin were analyzed by quantitative RT-PCR in NB15/FOXO3, NB4/FOXO3, and NB8/FOXO3 cells after treatment with 100 nM 4OHT for the times indicated. Statistical analysis was done by student’s t-test (***P<0.001, ***P*<0.01, *P<0.05).

**Supplemental Figure S4: Analyses of *BIM* promoter methylation and FOXO3 acetylation status.** (a) Genomic DNA was isolated from NB4/FOXO3, NB8/FOXO3, and NB15/FOXO3 cells treated with 50 nM 4OHT for 16 hours or 1 µM 5-azadC for 96 hours. Methylated DNA was quantified with MethyLight PCR based on TagMan® technology. Percentage of fully-methylated reference (PMR) values was calculated using *COL2A1* as reference gene. Bars represent ±s.e.m. of three independent experiments, each performed in triplicates. Significantly different to untreated ***P*<0.01, **P*<0.05. (b) Lysates of NB15, NB4, and NB8 cells were subjected to immunoprecipitation using anti-FOXO3 as precipitation antibody. Cleared cell lysates were analyzed for overall acetylation using an antibody against acetylated lysine. For control of precipitation efficiency FOXO3 amounts were stained. Tubulin served as loading control. Densitometry was performed using Labworks software version 4.5 (UVP, UK).

**Supplemental Figure S5: Prolin/Arginin polymorphism in the linker region between transactivation domain (TAD) and DNA-binding domain (DBD) of TP53.** cDNA was amplified from mRNA of untreated NB1, NB3, NB4, NB8, and NB15 cells and sequenced by Sanger sequencing demonstrating a base polymorphism at codon 72 changing proline to arginine. NB3 is homozygous for proline, NB4 is heterozygous whereas NB1, NB8, and NB15 are homozygous for arginine. Schematic structure of TP53 protein (GenBank: NP_000537.3): TAD, transactivation domain; TET, tetramerization domain with alignment of wild-type TP53 and TP53 expressed in NB4, NB8, and NB15 cells.

**Supplemental Figure S6: Differential regulation of FOXO3 target genes during etoposide-treatment in NB4, NB8, and NB15 cells.** NB4, NB8, and NB15 cells were treated for 0, 2, and 4 hours with 10 µg/ml etoposide and then subjected to RT-PCR to assess the levels of BIM, NOXA, and SESN3. Bars represent ±s.e.m. of three independent experiments, each performed in triplicate. Significantly different to untreated ***P<0.001, **P<0.01, *P<0.05; significantly different to NB15 etoposide treated ^#^P<0.05.

**Supplemental Figure S7:** NB1/Ctr and NB1/FOXO3 cells were treated for 72 hours with 50 nM 4OHT, before 0.8 µg/ml etoposide or 0.08 µg/ml doxorubicin were added for another 72 hours. Colonies were stained with crystal violet. Quantification was performed by photometric measurement after discoloration with 0.5% SDS in 50% ethanol. Shown are means ±s.e.m. of four independent experiments; **P<0.01, *P<0.05 (between ± 4OHT), ^###^P*<*0.001 ^##^P*<*0.01 (between 4OHT-treated cell lines).

**Supplemental Figure S8: Chemo-resistance by FOXO3 is regulated by TP53 and SESN3.** (a) SESN3 mRNA levels were assessed by quantitative RT-PCR in NB8/FOXO3-shCtr, NB8/FOXO3-shSESN3, NB4/FOXO3-shCtr, and NB4/FOXO3-shSESN3 cells. ***P<0.001 (b) ROS levels were analyzed in NB8/FOXO3-shCtr, NB8/FOXO3-shSESN3, NB4/FOXO3-shCtr, and NB4/FOXO3-shSESN3 cells after treatment with 10 µg/ml etoposide (two hours). Images were acquired by live-cell imaging using an Axiovert200M microscope with a 63x oil objective, bar 20 µm. Densitometric analyses were performed using AxioVision software version 4.8, *P<0.05. (c) Cell lysates of NB8/shCtr and NB8/shFOXO3-17 cells were subjected to immunoblot analyses using an antibody specific for FOXO3. GAPDH served as loading control.

**Methods exclusively used for supplemental data:**

***DNA methylation assay.*** Genomic DNA was isolated from 5 x 10^6^ cells treated with 4OHT and 5-azadC using PureLink®Genomic DNA Kit (Invitrogen, Carlsbad, USA) and modified with sodium bisulfite using EZ-DNA-Methylation-Gold Kit (Zymo Research, Irvine, USA) according to manufacturer’s instructions. *BIM*-promoter-methylation was quantified with MethyLight PCR based on TaqMan® technology as described previously.^2-4^ Primers and TaqMan probes are listed in Supplemental Table S4. Percentage of fully-methylated reference (PMR) values was calculated using *COL2A1* as reference gene.

Reference List

1. Nicoletti I, Migliorati G, Pagliacci MC, Grignani F, Riccardi C. A rapid and simple method for measuring thymocyte apoptosis by propidium iodide staining and flow cytometry. *J Immunol.Methods.* 1991; **139:** 271-9.

2. Eads CA, Danenberg KD, Kawakami K, Saltz LB, Blake C, Shibata D et al. MethyLight: a high-throughput assay to measure DNA methylation. *Nucleic Acids Res.* 2000; **28:** 1-8.

3. Ratzinger G, Mitteregger S, Wolf B, Berger R, Zelger B, Weinlich G et al. Association of TNFRSF10D DNA-methylation with the survival of melanoma patients. *Int J Mol Sci.* 2014; **15:** 11984-95.

4. Geiger K, Hagenbuchner J, Rupp M, Fiegl H, Sergi C, Meister B et al. FOXO3/FKHRL1 is activated by 5-aza-2-deoxycytidine and induces silenced caspase-8 in neuroblastoma. *Mol Biol Cell.* 2012; **23:** 2226-34.
