# Supplementary material for: FOXO3-mediated chemo-protection in high-stage neuroblastoma depends on wild-type TP53 and SESN3
Source: Oncogene. 2017 Sep 4;36(44):6190–203. doi: 10.1038/onc.2017.288 (PMC5671944; doi:10.1038/onc.2017.288)
Supplement: Supplementary Tables [file onc2017288x3.docx]

**Supplemental Table S1: List of primary antibodies**

| **Antibodies** | **Vendor** | **Catalog number** | **kDa** | **dilution** |
| --- | --- | --- | --- | --- |
| Acetyl_lysine | Cell Signaling Technology Inc., Boston, USA | #9441 | --- | 1:1000 |
| BCLXL | Cell Signaling Technology Inc., Boston, USA | #2762 | 30 | 1:500 |
| BIM | Becton Dickinson, Heidelberg, Germany | 559685 | 25/23 | 1:500 |
| BIM | Cell Signaling Technology Inc., Boston, USA | #2933 | 25/23 | 1:500 |
| FOXO3 | Cell Signaling Technology Inc., Boston, USA | #2497 | Endo 82/ectop 110 | 1:500 |
| GAPDH | Novus Biologicals, Littleton, USA | NB300-327 | 38 | 1.4000 |
| NOXA | Alexis Biochemicals, San Diego, USA | ALX-804-408 | 11 | 1:500 |
| P21CIP1 | Becton Dickinson, Heidelberg, Germany | 556430 | 21 | 1:500 |
| P27KIP1 | Becton Dickinson, Heidelberg, Germany | 554069 | 27 | 1:500 |
| SESN3 | Abcam, Cambridge, UK | Ab88454 | 57 | 1:500 |
| survivin | R&D Systems, Abingdon, UK | AF886 | 16 | 1:2000 |
| TP53 | Cell Signaling Technology Inc., Boston, USA | #9282 | 53 | 1:500 |
| α-Tubulin | Cell Signaling Technology Inc., Boston, USA | #3863S | 60 | 1:4000 |

**Supplemental Table S2: List of primers for chromatin-immunoprecipitation**

| **Gene** | **Forward primer sequence (5'-3')** | **Reverse primer sequence (5'-3')** |
| --- | --- | --- |
| BIM | GCCGCGCTGGAGTTACAAACTC | TGACTTCCCGGGGTTAGGTAGGAC |
| NOXA | CATTTCCCTTCCCTGTTACTGCCC | GGCGGGAGGGGAAGGGTTTA |
| SESN3 | GACTGAGGTGGATGTGGGTTATTCCTC | CATTCCCTTCCCCATCCCTCTC |

**Supplemental Table S3: List of primers for quantitative real-time PCR**

| **Gene** | **Forward primer sequence (5'-3')** | **Reverse primer sequence (5'-3')** |
| --- | --- | --- |
| BCLXL | ATGACCACCTAGAGCCTTGGATC | TCAGGAACCAGCGGTTGAAG |
| BIM | AGCACCCATGAGTTGTGACAAATC | CGTTAAACTCGTCTCCAATACGC |
| BIRC5 | CCACTGAGAACGAGCCAGACTTG | AGAAAGGAAAGCGCAACCGG |
| NOXA | AGCAGAGCTGGAAGTCGAGTGTG | TGATGCAGTCAGGTTCCTGAGC |
| SESN3 | GAGGATGTTGACACAACCATGCTG | CCGCCAGTAACTATCATACATGCG |
| GAPDH | TGTTCGTCATGGGTGTGAACC | GCAGTGATGGCATGGACTGTG |

**Supplemental Table S4: Sequence of MethyLight Primers and TagMan® probes**

| **Gene** | **Forward primer sequence (5'-3')** | **Reverse primer sequence (5'-3')** |
| --- | --- | --- |
| BIM | GCGACGTACGCGACACAA | GCGTTTTTCGAGGTTTTATATCGT |
| BIM_02 | CTCTTTCCTAACAACCAACCGC | GGTTGGTATTCGTTGGGTCG |
| COL2A1 | TCTAACAATTATAAACTCCAACCACCAA | GGGAAGATGGGATAGAAGGGAATAT |
|  | **TaqMan® probe sequence (5'-FAM; 3'-BHQ1)** |  |
| BIM | ATCACAACCTCCGCTAACCCG |  |
| BIM_02 | CCAACCTCGACGAAACCTCCGCT |  |
| COL2A1 | CCTTCATTCTAACCCAATACCTATCCCACCTCTAAA |  |

**Supplemental Table S5: List of primers for p53 mutation analyses**

| **mRNA variant** | **Forward primer sequence (5'-3')** | **Reverse primer sequence (5'-3')** |
| --- | --- | --- |
| p53 1, 2, 3, 4, 8 | GGTGACACGCTTCCCTGGATTG | TGGCAGGGGAGGGAGAGATG |
| p53 5, 6, 7 | AACTCTGTCTCCTTCCTCTTCCTACAG | CCAAAACCCAAAATGGCAGG |
| **DNA sequencing** | **Primer sequence (5'-3')** |  |
| p53 variant 1, 2, 3, 4, 8 | GGTGACACGCTTCCCTGGATTG |  |
|  | CCTGTCATCTTCTGTCCCTTCCC |  |
|  | GCGTGTGGAGTATTTGGATGACAG |  |
|  | AAGCGAGCACTGCCCAACAAC |  |
